# Molecular Crowding Alters the Interactions of Polymyxin Lipopeptides within the Periplasm of *E. coli*: Insights from Molecular Dynamics

**DOI:** 10.1021/acs.jpcb.3c07985

**Published:** 2024-03-08

**Authors:** Iain P. S. Smith, Conrado Pedebos, Syma Khalid

**Affiliations:** 1 School of Chemistry, University of Southampton, Southampton SO17 1BJ, U.K.; 2 Programa de Pós-Graduação em Biociências (PPGBio), Universidade Federal de Ciências da Saúde de Porto Alegre—UFCSPA, Porto Alegre 90050-170, Brazil; 3 Department of Biochemistry, University of Oxford, Oxford OX1 3QU, U.K.

## Abstract

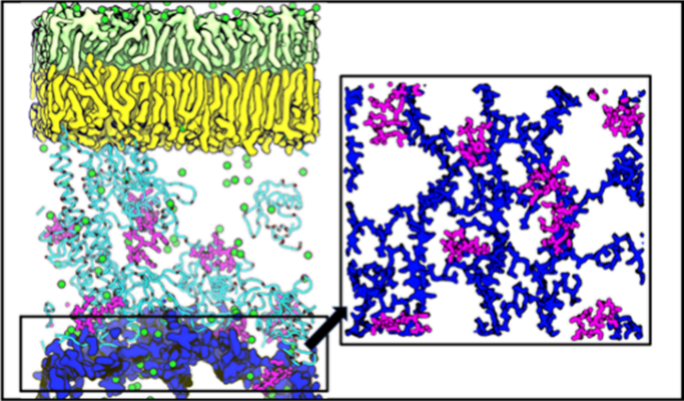

The cell envelope of Gram-negative bacteria is a crowded tripartite architecture that separates the cell interior from the external environment. Two membranes encapsulate the aqueous periplasm, which contains the cell wall. Little is known about the mechanisms via which antimicrobial peptides move through the periplasm from the outer membrane to their site of action, the inner membrane. We utilize all-atom molecular dynamics to study two antimicrobial peptides, polymyxins B1 and E, within models of the *E. coli* periplasm crowded to different extents. In a simple chemical environment, both PMB1 and PME bind irreversibly to the cell wall. The presence of specific macromolecules leads to competition with the polymyxins for cell wall interaction sites, resulting in polymyxin dissociation from the cell wall. Chemical complexity also impacts interactions between polymyxins and Braun’s lipoprotein; thus, the interaction modes of lipoprotein antibiotics within the periplasm are dependent upon the nature of the other species present.

## Introduction

Antimicrobial peptides (AMPs) are small, cationic membrane-active peptides. They are found in most living organisms, playing an important role in the innate immune response of their hosts.^1−3^ These peptides exhibit broad-spectrum antimicrobial activity against bacteria, fungi, and viruses,^4^ and thus they are of biomedical interest for use as therapeutic agents themselves or the inspiration for other novel antibacterial agents.

One class of highly potent AMPs are the polymyxins, a family of lipopeptides originally derived from the bacterial species *Paenibacillus polymyxa*.^5^ While there are five chemically distinct compounds within the family, namely, polymyxins A–E, only polymyxin B (PMB) and polymyxin E (PME or “colistin”) have been used in clinical practice. First approved for clinical use in the 1950s, their use was limited by the 1970s due to reports of severe nephro- and neurotoxicity.^6^ In recent decades, however, the emergence of multidrug resistant Gram-negative “superbugs” and their associated threat to global public health,^7^ coupled with improvements in clinical application^8^ and reports of lower levels of polymyxin toxicity,^9^ has led to the revival of their use as a last-resort intervention when all other treatment options have failed.^10,11^

**Figure 1:**
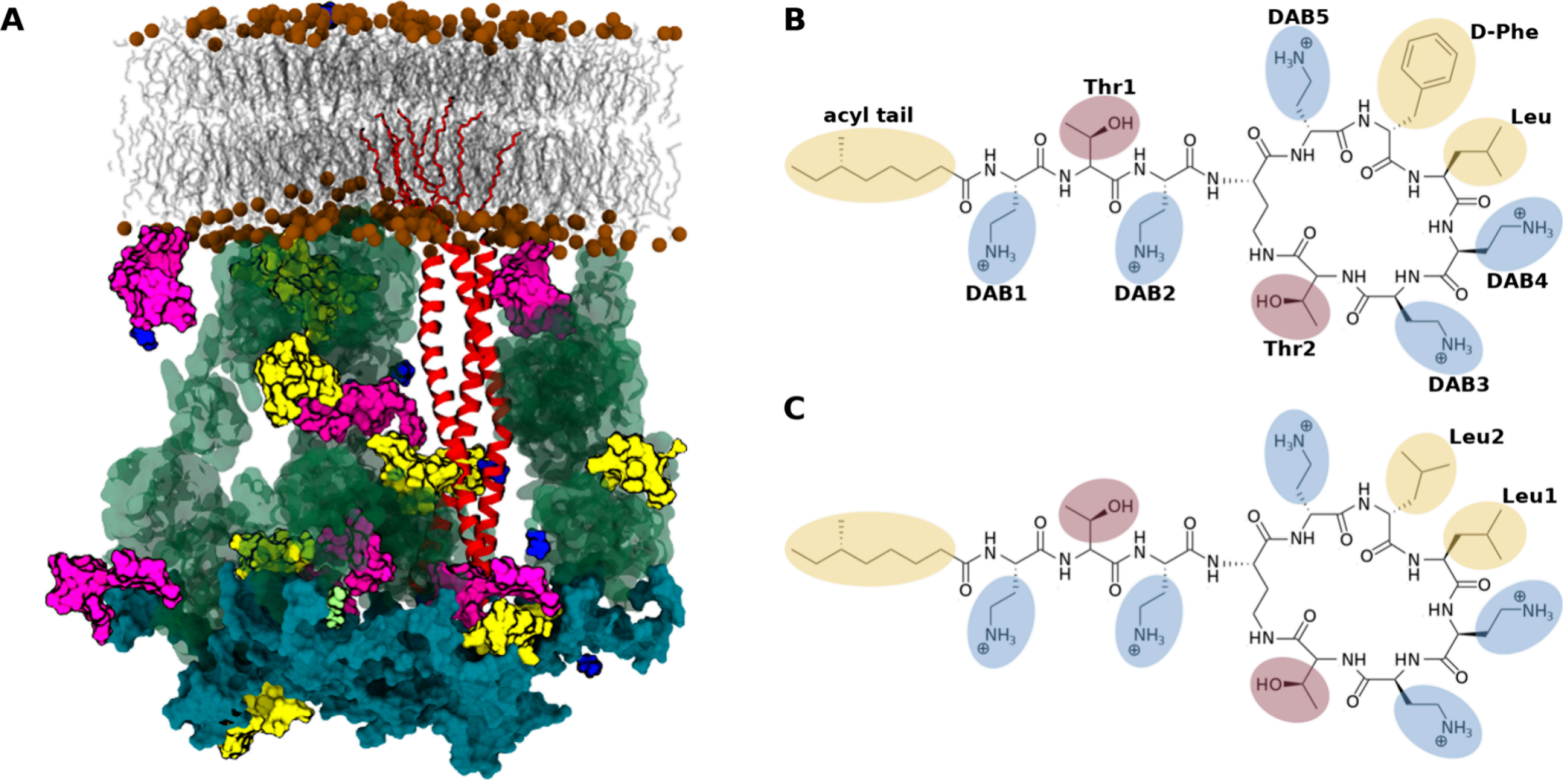
(A) Image of the cell envelope in the Ubiq crowding regime. Water and ions are omitted to aid visualization. Polymyxins are shown in yellow. OPG is in magenta, glycerol in blue, spermidine in lime green, ubiquitin in transparent green, BLP in red ribbons, and PGN in diffuse cyan. (B, C) Chemical structures of (B) PMB1 and (C) PME. Hydrophobic residues are highlighted in yellow. DAB is in blue and Thr in red.

Both PMB and PME are composed of a cyclic polypeptide ring with a branched fatty-acid tail. The chemical compositions of these molecules are almost identical, differing only by a single amino acid in the peptide ring, a phenylalanine in PMB being substituted for a leucine in PME (Figure 1B,C). Both molecules contain 5 noncyclized α,γ-diaminobutyric acid (DAB) residues that each carry a charge of +1 *e*, thus conferring a total charge of +5 *e* to the parent molecule. The amphipathic nature of polymyxins (due to cationic peptide portions and hydrophobic fatty-acid tails) enables them to disrupt bacterial and mammalian cell membranes,^12,13^ which is highly likely the origin of both their potent antimicrobial activity and clinical toxicity.

It is thought that polymyxins permeate across the bacterial outer membrane through a process of self-promoted uptake^14−18^ and that, subsequent to entry, they induce cell lysis by inserting into and disrupting the inner membrane.^14,17,19−21^ Relatively little is known, however, about the nature of their transport between the two membranes across the crowded aqueous periplasm of the Gram-negative bacterial cell envelope.

The bacterial cell envelope is a complex, multilayered structure that serves as a barrier between the interior of the cell and the often hostile external environment. In the case of Gram-negative bacteria such as *E. coli*, the cell envelope is composed of an inner membrane (IM) and outer membrane (OM) that form the boundaries of the crowded aqueous compartment known as the periplasm. Within the periplasm lies the cell wall, a mesh-like structure composed of cross-linked strands of peptidoglycan (PGN) polymers, along with a wide variety of proteins, osmolytes and ions.^22,23^ The only protein known to provide a covalent link with the cell wall is Braun’s lipoprotein (BLP, also known as “Lpp” or murein lipoprotein). BLP is anchored in the OM via a lipidated N-terminus, with the C-terminus being covalently bound to the peptide stem of a PGN monomer. With an estimated 10^5^ copies per cell, BLP is the most abundant protein in *E. coli* and acts as a structural scaffold linking the cell wall to the OM, maintaining their separation and facilitating the noncovalent interaction of other OM proteins with the cell wall.^24^ The cell envelope is thus a complex, crowded environment, and the considerable challenges posed to the movement of small molecules, such as antibiotics, throughout this space are beginning to be explored via *in vitro* and *in silico* studies.^25,26^

The recent emergence of bacterial strains resistant to both PMB and PME, coupled with the associated toxicities of their clinical use, necessitates either their modification or the development of completely novel antibiotics. As such, it is of immediate interest to establish a molecular-level understanding of each stage of the process via which they bring about cell death and, in particular, to begin to fill the gap in our understanding of how polymyxins are transported across the periplasm, toward the target of their antimicrobial function, the IM.

Previously we highlighted the promiscuity of PMB1 interactions with other molecular species within the periplasm of *E. coli*. In our study PMB1 was rarely uncomplexed throughout the simulations.^26^ From a mechanistic perspective, it is important to characterize whether molecular interactions experienced by PMB1 during translocation between the OM and IM, where action on the latter causes cell lysis, facilitate or hinder (if either) translocation. Furthermore, it is important to determine whether the same trends are observed for PME. It is thus prescient to investigate the comparative molecular interactions of PMB1 and PME within various models of the periplasm under different conditions of biomolecular crowding.

To this end, we have constructed a model of a portion of the *E. coli* cell envelope (Figure 1A) using the CHARMM36m force field. The model includes an asymmetric OM composed of LPS and phospholipids, a single-layered cell wall, and BLP. A series of molecular dynamics simulations (Table 1) of this model were performed in the presence of either PMB1 or PME (Figure 1B,C) under a range of periplasmic fluid compositions of differing complexity, we refer to these different periplasm compositions as “crowding regimes” from here on.

**Table 1:** Summary of All Simulations Performed for This Work^a^

crowding regime	polymyxin type	salt concn	osmolytes and ions	length (ns)
Poly	PMB1 (×8)	Neutralized	Cl^–^ (38)	3 × 250
		150 mM	Cl^–^ (247), K^+^ (209)	3 × 250
	PME (×8)	Neutralized	Cl^–^ (38)	3 × 250
		150 mM	Cl^–^ (247), K^+^ (209)	3 × 250
Osmo	PMB1 (×8)	Neutralized	Cl^–^ (41), glycerol (17), spermidine (1), OPG (9)	3 × 250
		150 mM	Cl^–^ (243), K^+^ (202), glycerol (17), spermidine (1), OPG (9)	3 × 250
	PME (×8)	Neutralized	Cl^–^ (41), glycerol (17), spermidine (1), OPG (9)	3 × 250
		150 mM	Cl^–^ (243), K^+^ (202), glycerol (17), spermidine (1), OPG (9)	3 × 250
Ubiq	PMB1 (×8)	Neutralized	Cl^–^ (41), glycerol (17), spermidine (1), OPG (9), ubiquitin (11)	3 × 250
		150 mM	Cl^–^ (243), K^+^ (202), glycerol (17), spermidine (1), OPG (9), ubiquitin (11)	3 × 250
	PMB1 (×8)	Neutralized	Cl^–^ (41), glycerol (17), spermidine (1), OPG (9), ubiquitin (11)	3 × 250
		150 mM	Cl^–^ (243), K^+^ (202), glycerol (17), spermidine (1), OPG (9), ubiquitin (11)	3 × 250
Ubiq-divalent	PMB1 (×8)	150 mM	Cl^–^ (243), Ca^2+^ (101), glycerol (17), spermidine (1), OPG (9), ubiquitin (11)	1 × 250
Ubiq-extended	PMB1 (×8)	150 mM	Cl^–^ (243), K^+^ (202), glycerol (17), spermidine (1), OPG (9), ubiquitin (11)	1 × 250

a Bracketed numbers give the number of each molecule present within the specific simulation regime.

Our simulations show that in the absence of a diverse chemical environment both PMB1 and PME tend to bind rapidly and irreversibly to the cell wall, predominantly via polar interactions between the positive DAB residues of the polymyxins and the various carboxylate groups on the peptide stems of the cell wall. These interactions are shown to be disrupted by the presence of physiological salt concentrations or increased biomolecular crowding, allowing for the dissociation of the polymyxins from the cell wall and their subsequent interaction with the various other components of the cell envelope. We provide evidence that certain cations, osmolytes, and proteins contribute to the disruption of polymyxin–cell wall interactions by forming competing interactions with the carboxylate groups on the peptide stems of the cell wall, reducing the number of such interaction sites available to nearby polymyxin molecules. Finally, we predict how the specific residue interactions that give rise to the binding of the polymyxins to BLP differ between PMB1 and PME and, further, how the balance of hydrophobic and polar residue interactions that underpin their binding is affected by varying the chemical diversity of the simulation environment.

## Methods

### Envelope Model Construction

The *E. coli* cell envelope model was based on a composition validated in previous work published by our group.^24,27^

An asymmetric model of the OM with an outer leaflet composed entirely of the lipid A region of LPS and an inner leaflet composed of 90% 1-palmitoyl 2-cis-vaccenic phosphatidylethanolamine (POPE), 5% 1-palmitoyl 2-cis-vaccenic phosphatidylglycerol (POPG), and 5% 1-palmitoyl 2-cis-vaccenic 3-palmitoyl 4-cis-vaccenic diphosphatidylglycerol (PVCL2, also known as cardiolipin) was constructed. This OM model has been validated in previous studies.^28−31^

The 1.9 Å crystal structure of the BLP homotrimer (1EQ7) from *E. coli* was used.^32^ All three BLP helices were acylated at their C-termini using the CHARMM-GUI membrane builder tool.^33^ The resulting acylated homotrimer was then manually inserted into the inner leaflet of the OM using VMD.^34^ The combined OM/BLP system was then equilibrated to ensure the correct lipid packing around the newly inserted acyl tails.

A single layer model of the peptidoglycan (PGN) cell wall was generated as previously reported;^24,27^ this model was equilibrated alone in solution before being covalently bound to the aforementioned membrane-inserted BLP molecule. The N-terminus of one BLP monomer within the homotrimer was covalently bound to the C-terminus of a meso-DAP residue located on a non-cross-linked peptide stem within the cell wall. Avogadro^35^ was used to generate a structure of the region surrounding the amide linkage connecting BLP and PGN. This structure was then passed through the CHARMM-GUI ligand reader tool^36^ to generate bond parameters for the covalently bound region.

### Envelope System Preparation

Three cell envelope crowding regimes were used in our work, namely, the Poly, Osmo, and Ubiq regimes. The simplest of these, the Poly regime, contained polymyxin molecules alone in the periplasmic region between the OM and the cell wall. In addition to these polymyxin molecules, the Osmo regime contained a range of small osmolytes, namely, spermidine, glycerol, and osmoregulated periplasmic glucans (OPG). These osmolytes were selected based on their chemical diversity and abundance within the *E. coli* cell envelope. The concentrations of these molecules within the periplasm are either documented or estimated in the literature^37−40^ and are reproduced in our model: glycerol (36 mM), OPG (20 mM), and spermidine (3 mM). The Ubiq regime was the most compositionally complex system studied in this work and, along with the polymyxin molecules and osmolytes, included ubiquitin proteins. The number of proteins added into the periplasm was chosen to reproduce a crowding volume fraction of φ ∼ 0.21, as estimated from experimental studies.^38^ Simulations of all three crowding regimes were performed in the presence of either PMB1 or PME. Each regime was prepared under two different concentrations of KCl, with neutralizing counterions alone or with neutralizing counterions and an excess salt concentration of 150 mM. We refer to these as the “neutralized” and “concentrated” systems from here on. Triplicate replica simulations of all system compositions were prepared; a summary of all simulations performed can be found in Table 1.

The CGenFF protocol^41^ was used to generate parameters for PMB1, the CHARMM-GUI ligand reader tool was used to generate parameters for spermidine and glycerol, while the CHARMM-GUI glycan reader tool^42^ was used to generate those for OPG. The crystal structure of ubiquitin (1UBQ) was obtained from the RCSB database, determined at a resolution of 1.8 Å.^43^

### Simulation Protocols

Simulations were performed using the GROMACS 2020.1 and 2021.2 molecular dynamics packages,^44^ utilizing the CHARMM36m force field^45^ and TIP3P water model.^46^ Our previous study^26^ primarily utilized the united atom GROMOS54A7 force field, with a single comparative simulation performed using the all atom CHARMM36m force field. In this case, the united atom model was chosen for its slight increase in simulation speed; however, as the available computational power of both national and institutional supercomputing facilities has continued to develop, it was more practical and accurate for the work presented in this study to use the highest resolution model available, i.e., the all atom CHARMM36m force field. Since the CHARMM36m force field was specifically parameterized for use with the TIP3 water model, this choice of force field also motivated the choice of water model used throughout this work.

Simulations were divided into two parts: equilibration simulations in *NVT* and *NPT* ensembles lasting for 200 ps and 40 ns, respectively, and production simulations in the *NPT* ensemble, which ran for 250 ns. A constant temperature of 310 K was maintained using the velocity rescale thermostat^47^ with a time constant of 1 ps. This choice of thermostat ensures the correct kinetic energy distribution of a system, thus reproducing the canonical (*NVT*) ensemble during simulation. The pressure was maintained anisotropically at 1 atm using the Parrinello–Rahman barostat^48^ with a time constant of 1 ps. This choice of barostat, combined with the velocity rescale thermostat, reproduces the biochemically relevant isothermal–isobaric (*NPT*) ensemble during simulation, thus ensuring the accuracy of the thermodynamic analysis. Hydrogen bonds were constrained using the LINCS algorithm.^49,50^ Stable treatment of these constraints required the use of a 1 fs integration time step. Long-range electrostatics were treated using the particle mesh Ewald method.^51^ The short-range electrostatic and van der Waals cutoffs were both set to 1.2 nm.

For the replicates, new initial configurations of all polymyxin, osmolyte, and ubiquitin molecules were generated, along with the resolvation and ionization of each system before being passed through the equilibration and production simulation phases. The initial velocities of all atoms were modified between each replicate at the start of *NVT* equilibration to ensure an unbiased sampling of the simulation phase space.

A recent review of the role of the Gram-negative outer membrane potential on the permeability of antibiotics discussed the importance of asymmetric ion concentrations to the propensity for certain antibiotics to accumulate within the periplasm.^52^ The model presented here comprises only a single membrane, and thus, due to the application of periodic boundary conditions, ions are free to move across the regions on either side of the membrane. Since the net flux of ions between the periplasmic and extracellular regions acts to negate the overall potential difference between these regions *in vivo,* an equilibrium condition of zero potential difference across the OM was chosen in our simulations as a best approximation.

Analyses were performed with scripts written using MDAnalysis,^53,54^ Gromacs utilities, and VMD. Kernel density estimate (KDE) curves were calculated using the Seaborn python package.^55^ The trend in interactions with the cell wall oxygens was calculated by considering the number of cell wall oxygen contacts (atomic separation of <4 Å) with K^+^ ions and the mean number of coincident cell wall oxygen contacts with the DAB residues of both peptides.

Linear regression models were fitted against the mean number of DAB residue contacts with cell wall oxygens, as a function of the number of coincident K^+^contacts with cell wall oxygens; this analysis was performed using the one-dimensional polynomial fitting algorithm provided by the Numpy^56^ python package. Confidence intervals were calculated, at a confidence level of 95%, against each complete set of DAB–cell wall oxygen contact counts corresponding to unique values of coincident K^+^–cell wall oxygen contacts, according to the standard form for the confidence interval.^57^

## Results

We performed a preliminary analysis of the number of hydrogen bonds formed between the cell wall and water molecules throughout each replicate simulation of the neutralized Poly and concentrated Ubiq regimes, representing, respectively, the most compositionally simple and complex systems simulated in this work. All three replicates of the concentrated Ubiq regime exhibited consistently fewer TIP3-PGN hydrogen bonds during the final 125 ns of simulation than were observed in any of the three replicates of the neutralized Poly regime during the same period (Figure S1).

The observed decrease in the availability of TIP3-PGN hydrogen bonding under a more complex chemical environment is indicative of the propensity for the various ions, osmolytes, and crowding proteins to interact with the cell wall, coating its surface and decreasing the number of available hydrogen bonding sites for the surrounding water molecules. This effect is likely to also impact the nature of interactions between the cell wall and polymyxin molecules, as the presence of an abundance of other biomolecules forces the polymyxins to compete for interaction sites on the cell wall surface.

Consideration of the simple system alone is therefore unlikely to be indicative of the behavior of the system *in vivo*, and thus, in conjunction with the more detailed analysis that follows, this result begins to highlight the importance of considering the true biological complexity of a system when determining the nature of biomolecular interactions through simulation.

### Polymyxin Interactions with the Cell Wall

**Figure 2:**
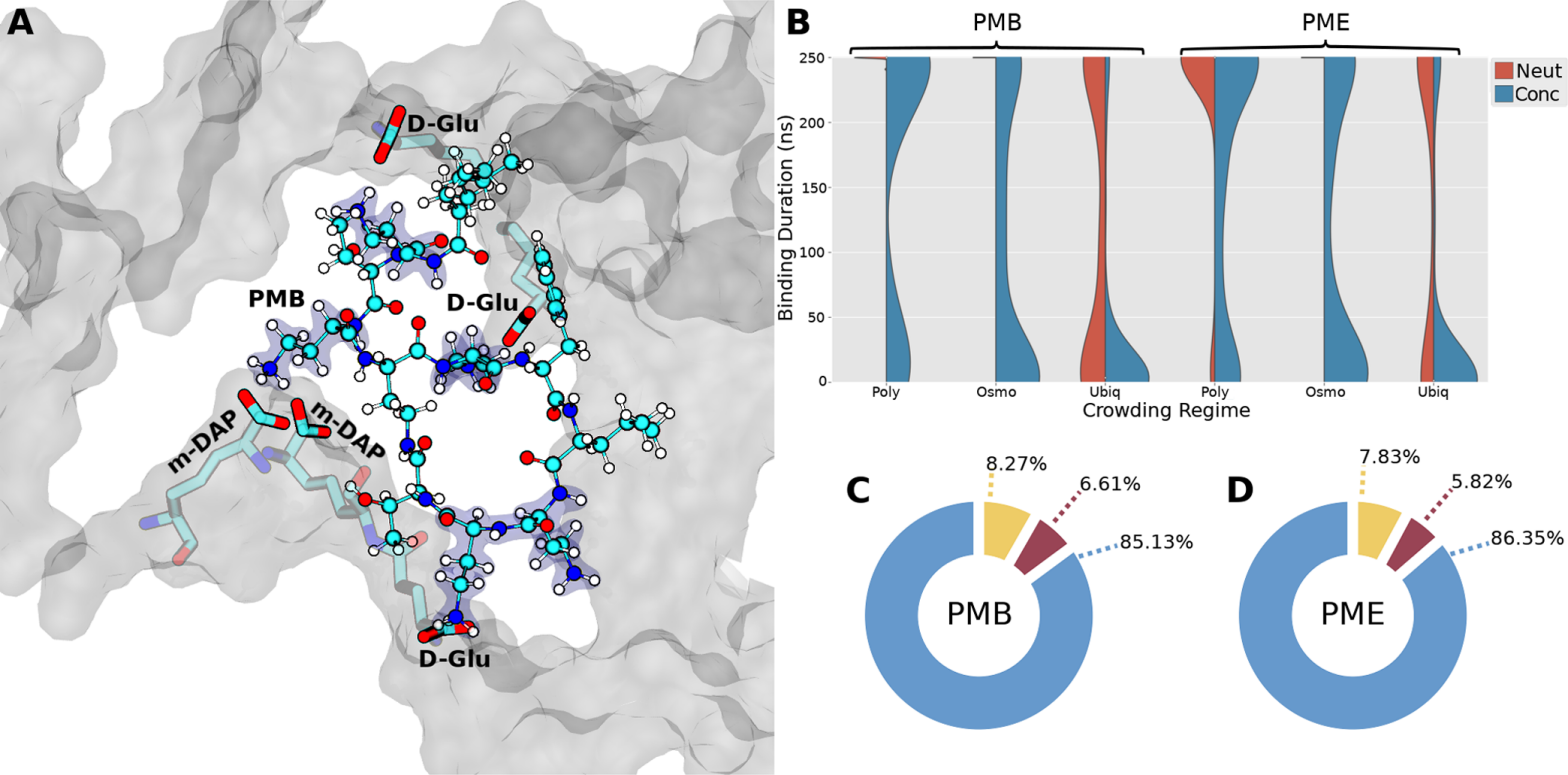
(A) Image of the PMB1 molecule inserted into a pore in the cell wall. Specific cell wall residues coordinated with PMB1 are shown in semitransparent licorice. Cell wall is shown as a transparent surface, with PMB1 in CPK. DAB residues of PMB1 are highlighted in blue bubbles. (B) Kernel density estimate (KDE) curves fitted to the binding durations of all unique instances of binding between polymyxin molecules and the cell wall. (C, D) Pie charts of residue interaction types between the cell wall and PMB1 (C)/PME (D), aggregated across all simulation regimes. Interactions involving the DAB/Thr/hydrophobic residues of polymyxins are colored in blue/red/yellow, respectively.

Focusing first on the nature of binding between the two polymyxin species and the cell wall, our analysis was split into two components: the duration of binding between the polymyxins and the cell wall and the biochemical nature of their interaction. Kernel density estimates (KDEs) were fitted to the observed binding durations across all replicates of each system and are presented in Figure 2B. The specific residue interactions were categorized according to interaction type (i.e., involving either hydrophobic, DAB, or Thr residues of the polymyxins), and the aggregated results for each polymyxin species across all simulation regimes are presented in Figure 2C,D. The complete data set for each simulation regime can be found in the Supporting Information (Tables S1 and S2).

### PMB1

Considering our analysis of interactions between PMB1 and the cell wall, we see that the chemical complexity of the simulation environment had a clear effect on the duration of their interactions (Figure 2B). Across all replicate simulations of the neutralized Poly regime, all but one PMB1 molecule were bound to the cell wall at the onset of production MD. These molecules bound to the cell wall during the first 10 ns of equilibration and did not dissociate from the cell wall for the duration of the subsequent production MD simulations. The anomalous PMB1 bound to the cell wall within 10 ns of production MD and thereafter also remained associated with the cell wall. A similar result was observed in the neutralized Osmo regime; all PMB1 molecules were initially bound to the cell wall and remained there for the duration of the simulation.

When these systems were simulated under concentrated conditions, multiple PMB1 molecules in all replicate simulations of both the Poly and Osmo regimes were not bound to the cell wall at the onset of production MD. Dissociation of PMB1 molecules from the cell wall was prevalent in both regimes and resulted in an abundance of short (<50 ns) and intermediate (50–200 ns) duration interactions between PMB1 and the cell wall. The addition of osmolytes increased the preference for short and intermediate duration interactions between PMB1 and the cell wall. Interactions persisting for less than 200 ns accounted for 39.4% of the total number of PMB1–cell wall interactions in the concentrated Poly regime, compared to 69.4% in the concentrated Osmo regime. This effect is shown explicitly in Figure 2B where a comparison of the KDE curves for the two regimes shows that the concentrated Osmo regime exhibits a higher relative density of shorter duration interactions.

Increased periplasmic crowding also impacted the interactions between PMB1 molecules and the cell wall under both neutralizing and excess salt concentrations. In the neutralized Ubiq regime, multiple PMB1 molecules in each replicate simulation were not bound to the cell wall at the onset of production MD, contrary to the behavior observed in the less crowded neutralized systems. Furthermore, of all the neutralized simulations reported in this study, dissociation of PMB1 from the cell wall was observed only in these, the most crowded, systems. The median PMB1–cell wall binding duration in the neutralized Ubiq regime was ∼96 ns, compared to just ∼1 ns in the concentrated Ubiq regime. This dramatic decrease is indicative of a strong preference for PMB1 to form short duration interactions with the cell wall under higher salt concentration; indeed, 87% of PMB1–cell wall interactions were classified as short (<50 ns) in the concentrated Ubiq regime, compared to just ∼43% under neutralized conditions. Notably, this result also indicates that the concentrated Ubiq regime (i.e., the most compositionally complex and crowded system) exhibits the largest proportion of short and intermediate duration interactions of any simulated system. This follows the observed trend from the concentrated Poly and Osmo regimes, whereby the addition of osmolytes led to a relative increase in the number of short and intermediate duration interactions, indicating that the presence of crowding ubiquitin proteins further enhances the effects caused by the inclusion of osmolytes.

### PME

PME molecules in both the neutralized Poly and Osmo regimes exhibited a preference for long duration (>200 ns) interactions with the cell wall; with both systems exhibiting median binding durations of 250 ns. Indeed, all PME molecules in these two systems were found to be bound to the cell wall at the beginning and end of production MD. In the neutralized Osmo regime, no dissociation of PME from the cell wall was observed in any of the replicate simulations. In one replicate of the neutralized Poly regime, however, a single PME molecule was seen to dissociate from the cell wall after 1.6 ns of production MD. This molecule was situated on the IM-facing surface of the cell wall at the onset of production MD, having passed through a pore in the cell wall during the equilibration process. After moving across the cell wall surface for approximately 26 ns, it bound to a non-cross-linked meso-DAP residue on the cell wall where it remained for the rest of the simulation.

The similarity of the KDE curve profiles for equivalent systems of PME and PMB1 in the Poly and Osmo regimes highlights that the effects of an increase in the salt concentration on the binding of PME to the cell wall were similar to those discussed for PMB1 (Figure 2B). Multiple PME molecules in each replicate of the concentrated Poly and Osmo regimes were not bound to the cell wall at the onset of production MD, and dissociation of PME from the cell wall was observed in all simulations of these regimes. Furthermore, interactions persisting for less than 200 ns accounted for 51.4% of PME–cell wall interactions in the concentrated Poly regime, compared to 65.1% in the concentrated Osmo regime, highlighting that, similar to the behavior observed in systems containing PMB1, the inclusion of osmolytes under concentrated conditions increases the preference for short and intermediate duration interactions between PME and the cell wall.

Increased periplasmic crowding further impacted the interactions between PME and the cell wall under both neutralizing and excess salt concentrations. In the neutralized Ubiq regime, at least one PME molecule in each replicate simulation was not bound to the cell wall at the onset of production MD. Furthermore, dissociation of PME from the cell wall under neutralized conditions was most prevalent in these most crowded systems, with a mean ∼1.4 instances of PME–cell wall binding per polymyxin in the Ubiq regime, compared ∼1.1 and 1.0 instances per polymyxin in the Poly and Osmo regimes, respectively. The median PME–cell wall binding duration in the neutralized Ubiq regime was ∼210 ns, compared to just ∼1 ns in the concentrated Ubiq regime. This dramatic decrease is indicative of a strong preference for PME to form short duration interactions with the cell wall under higher salt concentration; indeed, ∼81% of PME–cell wall interactions were classified as short (<50 ns) in the concentrated Ubiq regime, compared to just ∼38% under neutralized conditions. These results indicate that increases in the chemical complexity and crowding of the simulation environment affect compounding effects on the behavior of PME, disrupting PME–cell wall interactions and thus leading to a relative increase in the number of short and intermediate duration interactions, closely following the behavior observed in simulations of PMB1.

### Biochemical Nature of Cell Wall–PMB1/PME Interactions

Analysis of the biochemical nature of the interactions between the two polymyxin species and the cell wall showed that their binding was underpinned predominantly by interactions between the charged DAB residues of the polymyxins and the polar residues of the cell wall. This trend was consistent across all of the simulated systems. Cell wall interactions involving the DAB residues of polymyxins accounted for 85.4 ± 1.1% and 86.8 ± 2.0% (mean and standard deviation) for PMB1 and PME respectively. The polar meso-DAP, d-Glu, and d-Ala residues on the peptide stems of the cell wall were most prevalently involved in these interactions for both peptides (∼32%, ∼23%, and ∼19% respectively). Precise details are given in the Supporting Information (Tables S1 and S2). These results indicate that PMB1/E preferentially bind to the peptide region of the cell wall via polar interactions involving their cationic DAB residues (Figure 2A).

### Cationic Disruption of Polymyxin–Cell Wall Binding

**Figure 3:**
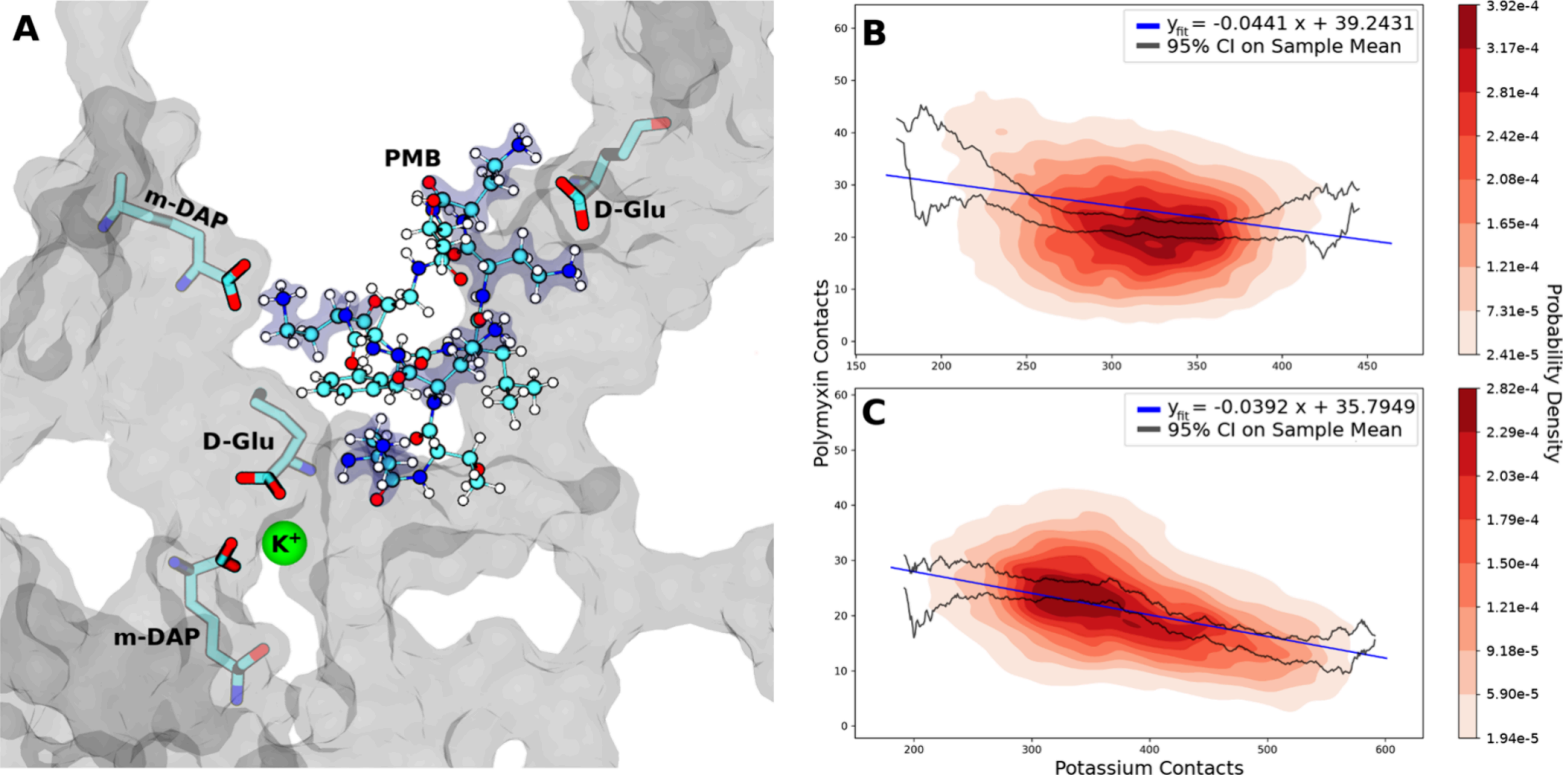
(A) Potassium cation coordination with cell wall carboxylate groups in the proximity of a PMB1 molecule. Representations are the same as in Figure 2. (B, C) Linear regression and probability densities fitted to coincident contact counts for potassium ions and (B) PMB1 or (C) PME with all cell wall oxygens. Confidence intervals were calculated using the standard error of the mean. Data were included from all three replicas of the concentrated Poly regime.

Next the biochemical origin of the disruption of cell wall–polymyxin binding upon increased system complexity and crowding was investigated. The meso-DAP, d-Glu, and d-Ala residues of the cell wall each contain anionic carboxylate groups that readily form salt bridges with the cationic DAB residues of the polymyxins in the MD simulations (Figure 2A). This polymyxin–carboxylate interaction has previously been reported from studies of nanoparticles decorated with carboxylate groups.^58^ Within the concentrated simulation regimes, K^+^ ions also coordinated to these cell wall carboxylate groups. In some cases, this coordination occurred in close proximity to polymyxin molecules that were already bound to the cell wall, leading to direct competition between the cationic groups to form salt bridges with the carboxylate groups (Figure 3A). We note here K^+^ ions are not present in the neutralized simulation regimes, and therefore, no such coordination was observed in any of the neutralized simulations.

The number of contacts (separation <4 Å) between cell wall oxygen atoms and polymyxin DAB residues vs cell wall oxygen atoms and K^+^ ions was compared for the concentrated Poly simulations. A negative linear correlation was calculated for both PMB1 (Figure 3B) and PME (Figure 3C), supporting the idea that K^+^ ions compete with polymyxin molecules for carboxylate interaction sites on the cell wall.

**Figure 4:**
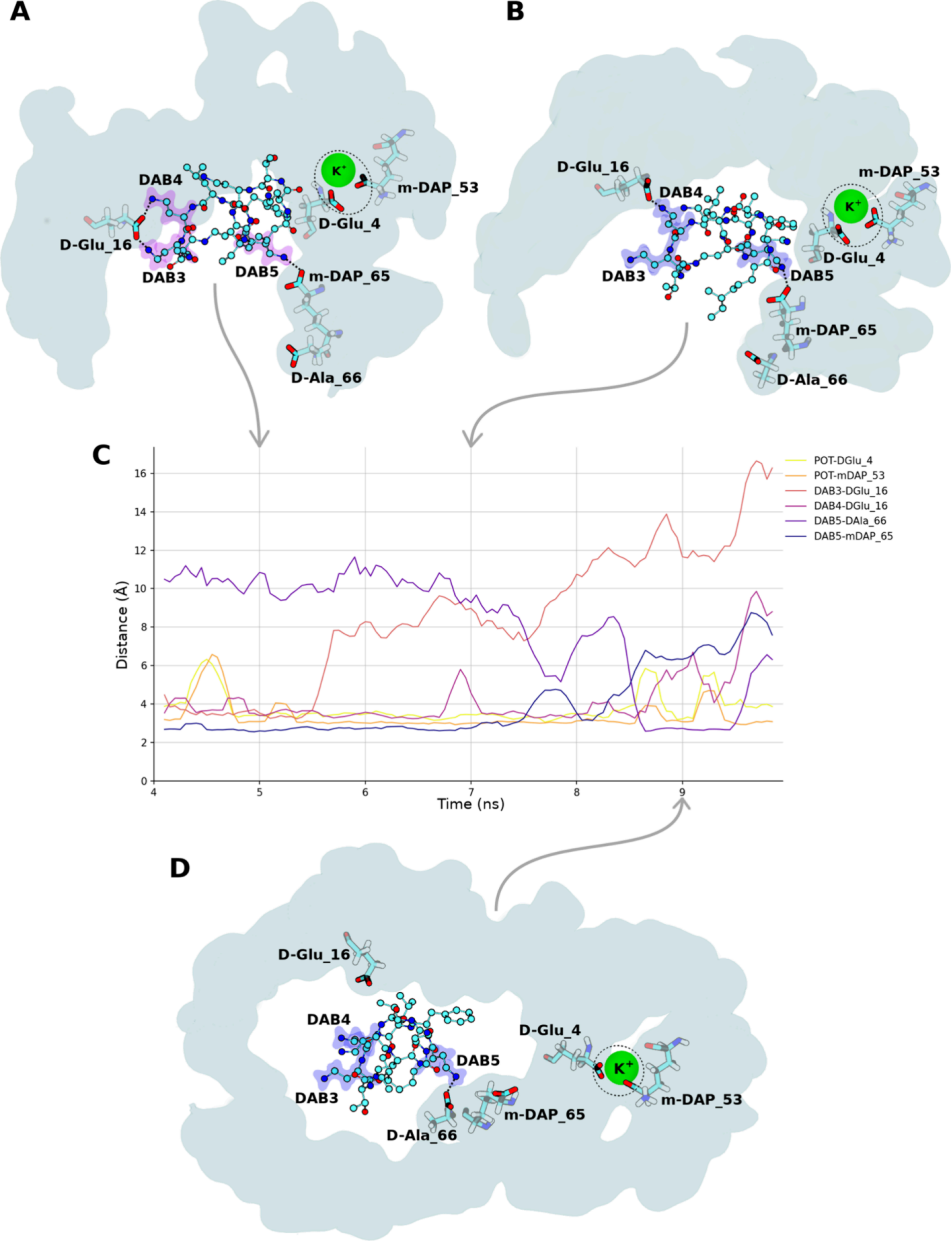
Time series of polymyxin–cell wall binding disruption resulting from the proximal binding of K^+^ during one simulation of the concentrated Poly regime. (A) Initial binding location of PMB1, 2.5 ns after initial contact between K^+^ and the cell wall. (B) First transition state of PMB1. (C) Hydrogen bond distances between key residue interactions underpinning the coordination of PMB1/K^+^ with the cell wall. D) Final transition state of PMB1. PMB1 is represented by Goodsell CPK, K^+^ in green vdW, interacting cell wall residues in transparent Goodsell licorice, and the cell wall surface in solid cyan. Dotted lines highlight specific hydrogen bonds, and dotted circles highlight K^+^–cell wall coordination. PMB1 hydrogens have been omitted for visual clarity.

Examples of the binding of K^+^ to the anionic residues of the cell wall, shown in Figure 3A, were observed throughout the simulations in which K^+^ ions were present. The process of competitive binding to the cell wall between K^+^ and PMB1 can be exemplified by considering the specific scenario below (Figure 4). Prior to the binding of K^+^ to the cell wall, PMB1 was bound to the cell wall via 3 distinct hydrogen bonds: DAB4-DGlu16, DAB3-DGlu16, and DAB5-mDAP65 (Figure 4A). Approximately 3 ns after K^+^ bound to the cell wall, a disruption of the DAB3-DGlu16 interaction was observed (Figure 4B). The dissociation of this hydrogen bond resulted in increased mobility of the PMB1 molecule, highlighted by the subsequent brief dissociation of the DAB4-DGlu16 and DAB5-mDAP65 interactions (Figure 4C). Approximately 6 ns after K^+^ bound to the cell wall, the DAB4-DGlu16 and DAB5-mDAP65 interactions were permanently disrupted and replaced by the DAB5-DAla66 hydrogen bond (Figure 4D). The DAla66 residue of the cell wall was located further from the K^+^ ion than the mDAP65 residue, and so the transition of the DAB5 interaction between these residues represents a movement of the PMB1 molecule away from the cell wall bound K^+^. Dissociation of the DAB5-DAla66 interaction occurred ∼7 ns after the initial binding of K^+^ with the cell wall (Figure S2), with the complete dissociation of PMB1 from the cell wall occurring within a further 2 ns of simulation.

**Figure 5:**
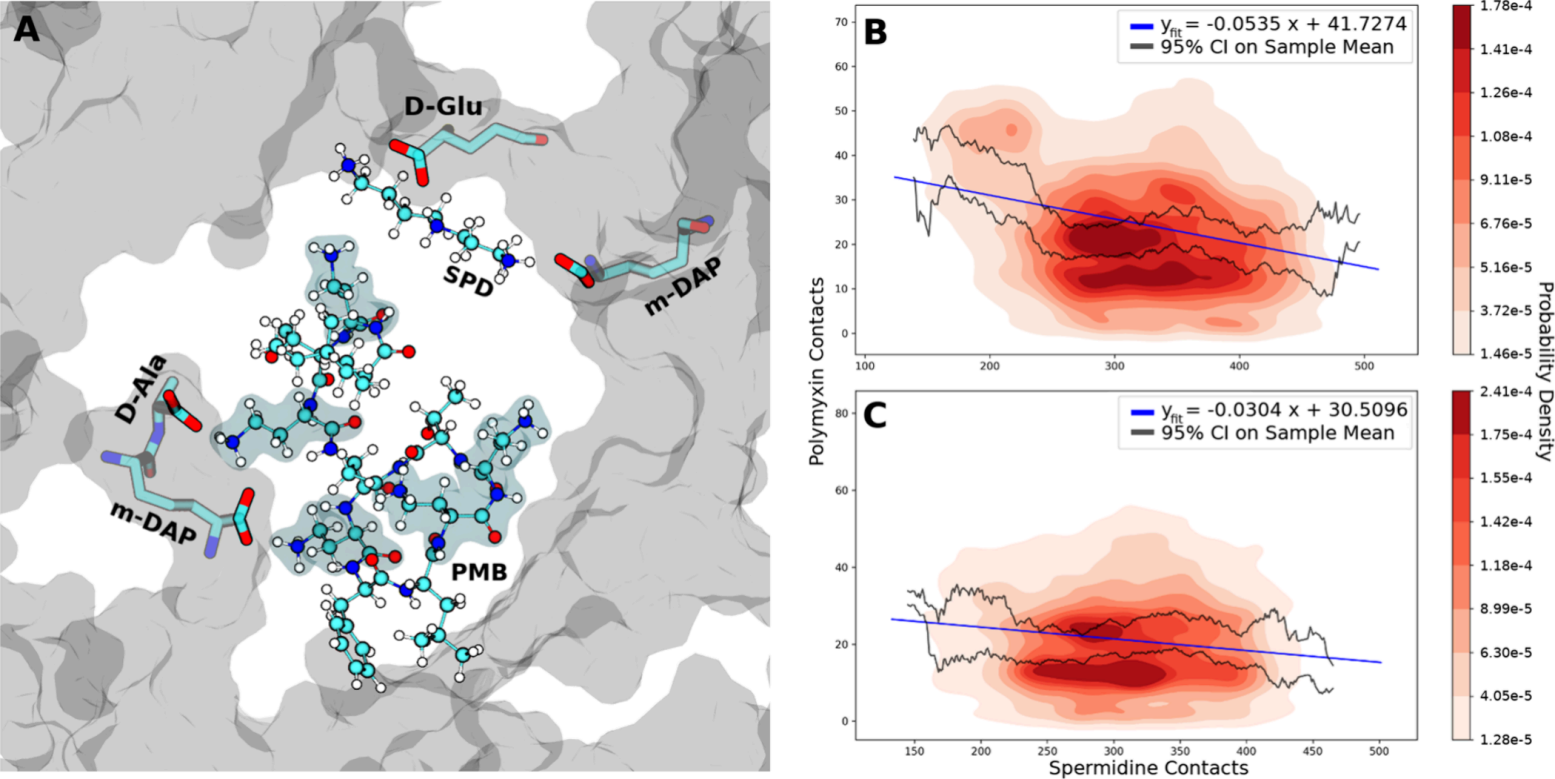
(A) Spermidine coordination with cell wall carboxylate groups in the proximity of a PMB1 molecule. Representations are the same as in Figure 2. (B, C) Linear regression and probability densities fitted to coincident contact counts for spermidine molecules and (B) PMB1 or (C) PME with all cell wall oxygens. Confidence intervals were calculated using standard error of the mean. Data were included from all three replicas of the concentrated Osmo regime.

Similar to K^+^ ions, spermidine was also observed to coordinate with the carboxylate groups on the cell wall peptide residues. This coordination was observed across all simulation regimes and occasionally occurred in close proximity to polymyxin molecules that were already bound to the cell wall (Figure 5A). A timeline of the residue interactions formed by PMB1 and spermidine with the cell wall during the example proximal binding event in Figure 5A is presented in the Supporting Information (Figure S3).

Given the generally linear structure of spermidine and its small size relative to the polymyxins, spermidine regularly inserted into the junction points of the cell wall pores inaccessible to polymyxin molecules (Figure S4). This behavior, alongside the concentration of spermidine being lower than other molecular components within our systems, resulted in rare observations of direct competition between polymyxins and spermidine for carboxylate interaction sites. Despite this, within the concentrated Osmo regime, a negative linear correlation was calculated between the number of cell wall oxygen contacts with spermidine molecules and the cell wall oxygen contacts with the DAB residues of both PMB1 (Figure 5B) and PME (Figure 5C).

The probability densities underlying these data do not provide a clear visual indication of this trend, and the presence of density clusters perhaps indicates an unidentified variable not accounted for in the linear regression model. However, the existence of such a negative correlation in conjunction with explicit observations of spermidine binding to cell wall carboxylate groups in close proximity to polymyxin molecules (Figure 5A) supports the idea that, similar to K^+^ ions, spermidine competes with polymyxin molecules for carboxylate interaction sites on the cell wall.

In earlier analysis of the binding durations of polymyxins with the cell wall it was shown that the presence of crowding ubiquitin proteins resulted in shorter duration interactions between the cell wall and PMB1, leading to the abundant dissociation of PMB1 from the cell wall under neutralized conditions. We next sought to ascertain whether ubiquitin may also be competing with PMB1 for cell wall carboxylate interaction sites.

**Figure 6:**
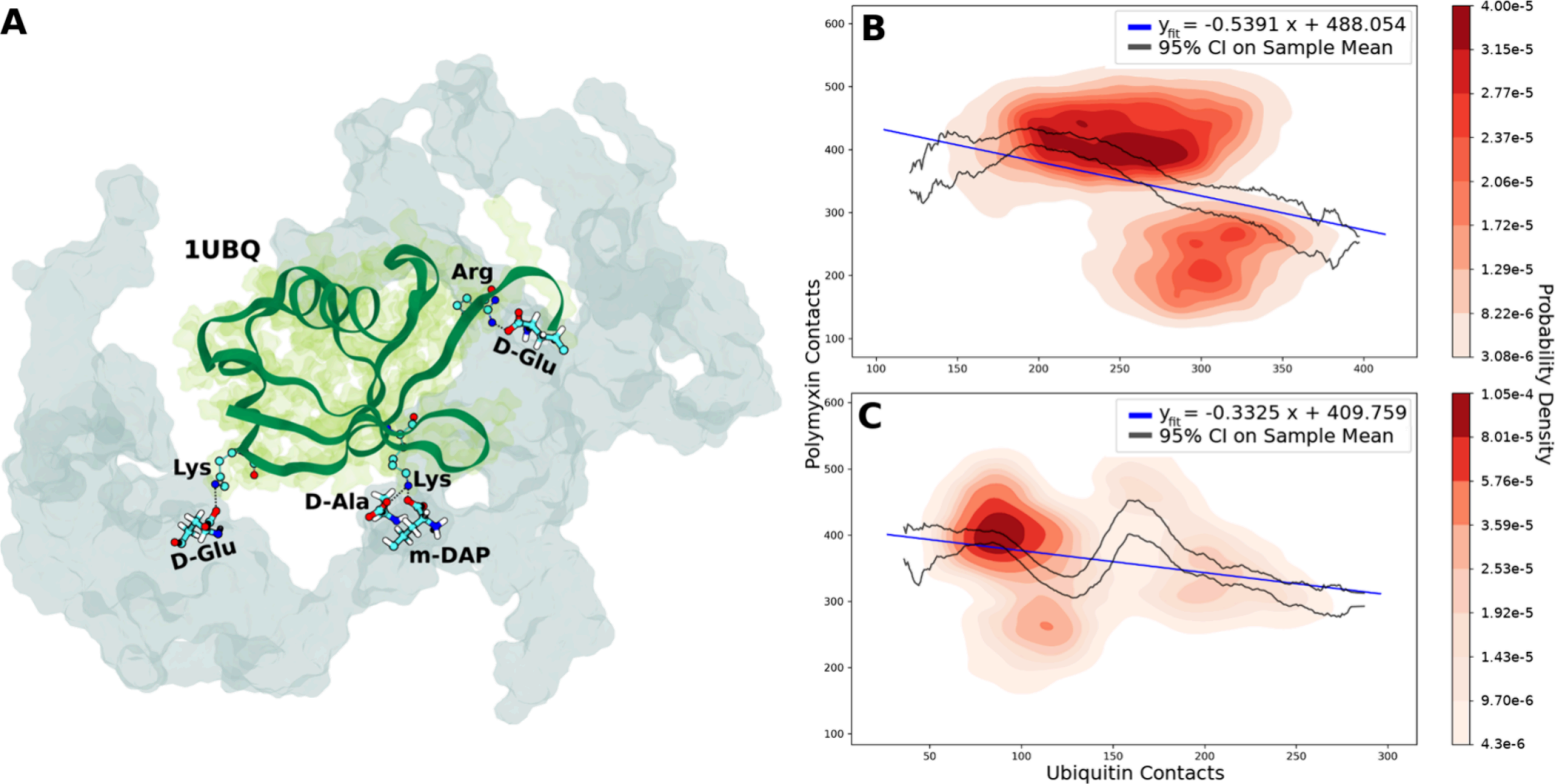
(A) Ubiquitin coordination with cell wall carboxylate groups. The cell wall is represented in transparent cyan, and ubiquitin is represented with green ribbons and a transparent green surface plot. (B, C) Linear regression and probability densities fitted to coincident contact counts for ubiquitin molecules and PMB1 with all cell wall oxygens in the (B) neutralized or (C) 150 mM concentration regimes. Confidence intervals were calculated using standard error of the mean. Data were included from all three replicas of the Ubiq regime under each concentration.

Similar to both K^+^ ions and spermidine, ubiquitin was also observed to interact with the various carboxylate groups of the cell wall peptide residues (Figure 6A), predominantly via basic Lys and Arg residues. These interactions were observed under both neutralized and concentrated ionic conditions. Due to the large size of ubiquitin and the relatively high concentration of the crowding protein within our systems, the interactions regularly occurred in close proximity to polymyxin molecules.

Negative linear correlations were calculated between the number of cell wall oxygen contacts with ubiquitin molecules and the mean number of coincident cell wall oxygen contacts with the DAB residues of PMB1 under both neutralized (Figure 6B) and concentrated (Figure 6C) ionic conditions. The probability densities underlying these two data sets show the formation of density clusters, more distinct than those observed in the spermidine data, perhaps indicating once more that there are unidentified variables not accounted for in our linear regression model. However, the overall negative correlation and explicit observation of ubiquitin molecules binding to the carboxylate groups on the cell wall peptide residues indicate that the cationic and amine-rich residues of ubiquitin, similar to potassium and spermidine, compete with polymyxin molecules for carboxylate interaction sites on the cell wall. This is likely to be generalizable for periplasmic proteins of similar size to ubiquitin which share surface residue characteristics.

### Polymyxin Diffusion

**Figure 7:**
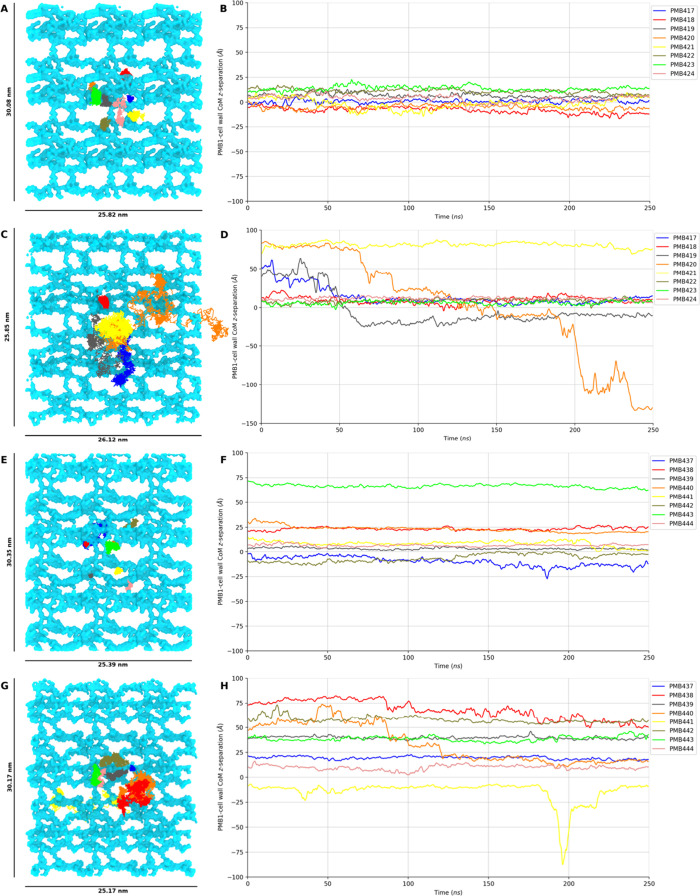
PMB1 diffusion in representative replica simulations of the neutralized Osmo (A, B), concentrated Osmo (C, D), neutralized Ubiq (E, F), and concentrated Ubiq (G, H) regimes. Plots on the left-hand side illustrate the trajectories of each PMB1 molecule in the *x*–*y* plane throughout each simulation. Plots on the right-hand side show the CoM *z*-coordinate of each PMB1 throughout each simulation.

Analysis of PMB1 diffusion in representative replica simulations of the Osmo and Ubiq regimes was also performed (Figure 7). Visual representation of PMB1 diffusion in the *x*–*y* plane (i.e., parallel to the surface of the cell wall) highlighted that PMB1 exhibited limited lateral diffusion within the neutralized crowding regimes (Figure 7A,E) as compared to the concentrated regimes (Figure 7C,G). In both the neutralized Osmo and Ubiq regimes, all PMB1 molecules remained localized around their initial *x*–*y* positions for the entire duration of simulation. In contrast, multiple PMB1 molecules in the concentrated Osmo and Ubiq regimes exhibited periods of free diffusion throughout the periplasm, indicating that the presence of K^+^ disrupted the constraints on lateral PMB1 diffusion. Furthermore, PMB1 molecules diffusing throughout the aqueous phase of the periplasm in the concentrated Osmo regime (PMB417, PMB419, and PMB420 in Figure 7C) exhibited a greater extent of lateral diffusion than those in the concentrated Ubiq regime (PMB438, PMB439, PMB440, PMB442, and PMB443 in Figure 7G), indicating that the presence of crowding ubiquitin proteins constrained the lateral diffusion of PMB1 molecules.

These results were mirrored for PMB1 diffusion in the *z* dimension in each regime. In the neutralized Osmo regime (Figure 7B) all PMB1 molecules had CoM *z*-coordinates within 25 Å of the cell wall CoM *z*-coordinate for the entire duration of simulation, indicative of the unbroken binding of polymyxins to the cell wall observed in this regime. In the neutralized Ubiq regime (Figure 7F), all but one PMB1 molecule remained localized around their initial *z*-positions for the entire duration of the simulation. The single anomalous PMB1 (PMB437 in Figure 7F) was initially bound to the cell wall at a *z*-position close to the cell wall CoM; it dissociated briefly from the cell wall after ∼180 ns before quickly (<5 ns) reassociating and remaining bound to the cell wall for the remaining duration of simulation. In this regime, PMB1 molecules that were not bound to the cell wall at the onset of simulation also remained constrained close to their initial *z*-positions (PMB443, PMB440, PMB438 in Figure 7F), highlighting the spatial constraints applied to PMB1 diffusion by the presence of crowding proteins. In contrast, polymyxin molecules in both the concentrated Osmo and Ubiq regimes were able to diffuse freely throughout the periplasm, reflected by the large changes in the CoM *z*-coordinate of multiple PMB1 molecules in both of these regimes (Figure 7D,H).

The diffusion analysis was repeated for one additional simulation of the concentrated Ubiq regime, which included divalent Ca^2+^ ions instead of K^+^. Under these conditions, all PMB1 molecules were observed to undergo periods of free diffusion throughout the periplasm, resulting in all PMB1 molecules exhibiting broad extents of lateral diffusion (Figure S5A). These results were mirrored during analysis of PMB1 *z*-axis diffusion, where PMB1 molecules were observed to freely dissociate from the cell wall, enabling their exploration of the full vertical domain of the periplasm (Figure S5B). Analysis of the distribution of Ca^2+^ ions within this regime indicated that Ca^2+^ coated the cell wall and outer leaflet of the OM, with a relative absence of Ca^2+^ in the rest of the periplasmic compartment (Figure S7). This is in contrast to the distribution of K^+^ ions which indicated that while K^+^ also tended to interact with the cell wall, they were present in abundance throughout the rest of the periplasmic region. Combined, these results illustrate that the interactions between the cell wall and both monovalent and divalent cations acted to disrupt the otherwise stable binding of polymyxins to the cell wall. This disruption increased with the charge of the cation and was correlated to the extent to which the cations preferentially bound to the cell wall.

### Polymyxin Interactions with BLP

**Figure 8:**
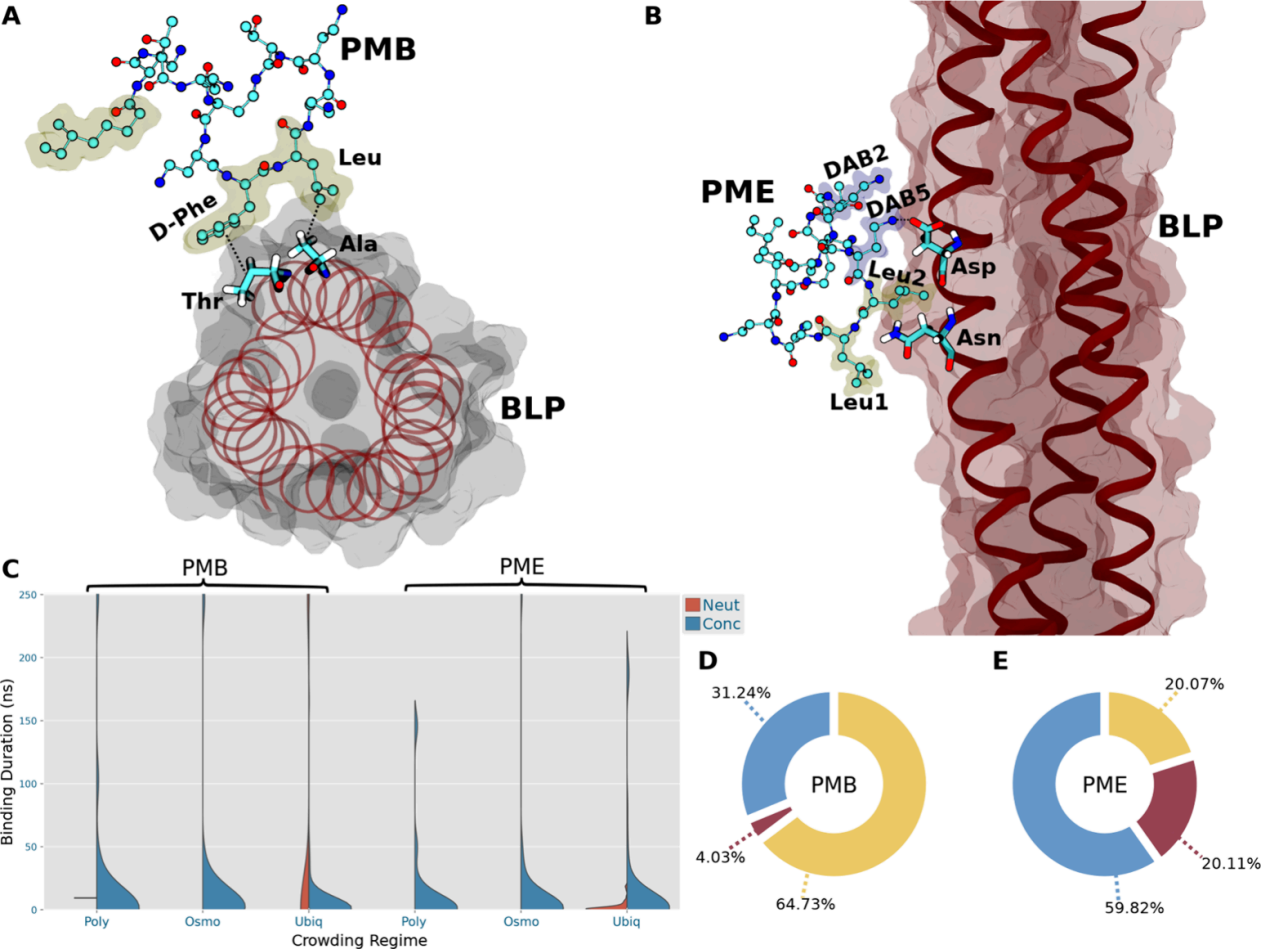
(A) PMB1 binding to BLP via the dominant d-Phe residue in the concentrated Osmo regime. Hydrophobic residues of PMB1 are highlighted with yellow bubbles. (B) PME binding to BLP via the Leu1/Leu2/DAB5 triad in the concentrated Osmo regime. Hydrogens were omitted for visual clarity. (C) Kernel density estimation (KDE) curves fitted to the binding durations of all unique instances of binding between polymyxin molecules and BLP. (D, E) Pie charts of residue interaction types between the cell wall and PMB1 (D)/PME (E) in the concentrated Osmo regime. Interactions involving the DAB/Thr/hydrophobic residues of polymyxins are colored in blue/red/yellow, respectively.

Next the binding between the two different polymyxin peptides and BLP was characterized. The analysis was again divided into two components: the duration of binding between the polymyxins and BLP, and the biochemical nature of these interactions. Kernel density estimates (KDEs) were fitted to the observed binding durations across all replicates of each system and are presented in Figure 8C. The specific residue interactions were categorized according to interaction type (i.e., involving either hydrophobic, DAB or Thr residues of the polymyxins) and the results for each polymyxin in the concentrated Osmo regime are presented in Figures 8D,E. The complete data set for all simulation regimes can be found in the Supporting Information (Tables S3 and S4).

### PMB1

Across all simulations of the neutralized Poly and Osmo crowding regimes, there was only one instance of PMB1 interaction with BLP. This interaction was observed in the Poly regime, in which one PMB1 was bound to BLP at the onset of production MD. This molecule immediately began moving along the surface of BLP toward the cell wall, making contact with the cell wall after ∼9 ns. After this, it dissociated from BLP and remained bound to the cell wall for the remaining duration of the simulation. This “walking” behavior of PMB1 along BLP has been reported in previous studies of similar systems.^26^ In contrast, in all three replicate simulations of the neutralized Ubiq crowding regime, some initially bound PMB1 molecules remained bound to BLP for the duration of simulation. Indeed, other than the single PMB1 molecule in the neutralized Poly regime, it was only in these most crowded simulations that PMB1 molecules were seen to interact with BLP under neutralized conditions, with 21 unique instances of PMB1-BLP binding observed in this regime.

Interactions between PMB1 and BLP were prevalent across all high salt concentration crowding regimes, with 26 unique PMB1-BLP interactions observed in both the Poly and the Osmo regimes and 110 in the Ubiq regime. There was a strong preference for short duration interactions in all cases, with median binding durations of 0.25, 1.00, and 0.90 ns for the Poly, Osmo, and Ubiq regimes, respectively. In contrast to this trend, one replicate of each regime contained a single PMB1 molecule that remained bound to BLP for the entire duration of the simulation.

It was previously reported that the serine and acidic residues of BLP have a particular propensity to interact with PMB1 due to their ability to form hydrogen bonds and salt bridges with the cationic DAB residues of PMB1.^26^ Across all of our PMB1 simulations, ∼40% of all observed residue interactions were formed between the DAB residues of PMB1 and the various polar residues of BLP. Interactions between hydrophobic residues of PMB1 and BLP accounted for ∼45% of observed residue interactions, with the remaining ∼15% formed between the Thr residues of PMB1 and the polar residues of BLP; equivalent to ∼27% of observed polar interactions. The serine residues of BLP interacted with both the DAB and Thr residues of PMB1, however, such interactions accounted for only ∼7.4% of the total number of residue interactions; a lower percentage than was observed for the Gln (∼17.5%), Asp (∼14.6%), Ala (∼14.0%), Arg (∼12.0%), Asn (∼10.5%), and Lys (∼8.8%) residues of BLP. It is evident, therefore, that while interactions with the acidic residues of BLP are important to the binding of PMB1, a more complete picture of their binding requires a balance of both electrostatic and hydrophobic interactions, involving many different residues.

We next consider the details of the interaction types in each simulation regime. In the concentrated Poly regime, polar interactions involving the DAB or Thr residues of PMB1 were the dominant component in PMB1-BLP binding, making up ∼59% of all observed residue interactions; the DAB residues were of particular importance, accounting for ∼42%. Despite this, the contribution of PMB1 hydrophobic residues to BLP binding was still significant, with the d-Phe, acyl tail, and Leu residues being responsible for, respectively, ∼15.8%, ∼15.2%, and ∼10% of observed residue interactions. If we consider the results in context of the location of each residue in the structure of PMB1, we find that ∼67.3% of interactions involved residues residing on the heptapeptide ring of PMB1, with interactions involving the branched fatty acid tail making up the remaining ∼32.7%. Of those residues that reside on the heptapeptide ring of PMB1, the hydrophobic d-Phe and Leu residues were responsible for a combined ∼25.8% of all residue interactions, the DAB3, DAB4 and DAB5 residues were responsible for ∼28.4%, and the Thr2 residue was responsible for ∼13.1%. These data imply that, within this regime, PMB1 preferentially bound to BLP via its heptapeptide ring, utilizing a variety of polar and hydrophobic interactions.

In the concentrated Osmo regime, hydrophobic interactions involving the acyl tail, Leu, and d-Phe residues of PMB1 dominated the binding with BLP, accounting for ∼65% of observed residue interactions, ∼24% greater than was observed in the concentrated Poly regime. The acyl tail and Leu residues of PMB1 were responsible for respectively ∼23.0% and ∼10.5% of observed residue interactions in the concentrated Osmo regime. The d-Phe residue was the largest single contributor, accounting for ∼31.3% of all interactions. Notably, the DAB5 residue alone accounted for a further ∼22.6% of residue interactions, with the other four DAB and two Thr residues cumulatively accounting for the final ∼12.7%. This result is of particular interest given that the d-Phe residue neighbors both the Leu and DAB5 residues within the heptapeptide ring of PMB1; this triad of residues therefore represents a combined ∼64.4% of all residue interactions, indicating that this specific region of PMB1 plays a crucial role in its binding to BLP. Thus, we find that the binding between BLP and PMB1 within this regime is underpinned primarily (∼87.4%) by interactions involving the acyl tail and the Leu/d-Phe/DAB5 triad of PMB1 (Figure S6).

Across all replicate simulations of the neutralized Ubiq regime, hydrophobic interactions accounted for ∼37.6% of observed residue interactions, with the d-Phe, acyl tail, and Leu residues contributing to, respectively, ∼15.3%, ∼13.1%, and ∼9.2%. The various charged and polar residues accounted for the remaining ∼62.4% of observed residue interactions, with the largest contributions coming from the Thr1 (∼20.1%), DAB5 (∼19.4%), DAB1 (∼9.1%), and DAB2 (∼9.0%) residues. Clustering these results based on the location of each residue within the structure of PMB1 highlights that the Leu/d-Phe/DAB5 triad was involved in a combined ∼43.9% of residue interactions, while the polar DAB1/Thr1/DAB2 residues of the branched fatty acid tail combined were involved in ∼38.1%. These two residue triads therefore accounted for ∼82.0% of all observed residue interactions in this regime, indicating that these regions of PMB1 play a crucial role in PMB1-BLP binding under these simulation conditions.

In the concentrated Ubiq regime, hydrophobic interactions were responsible for ∼48.0% of observed residue interactions. While this is a lower contribution than was observed in the concentrated Osmo regime (∼65%), it is considerably higher than the result obtained from the neutralized Ubiq regime (∼37.6%), indicating that the addition of an excess salt concentration to the Ubiq system exacerbates the involvement of the hydrophobic residues of PMB1 in binding with BLP.

The acyl tail (∼28.0%), DAB1 (∼17.5%), Leu (∼13.3%), DAB5 (∼10.1%), and Thr1 (∼10.0%) residues of PMB1 were the largest contributors, accounting for a combined ∼79% of observed residue interactions in the concentrated Ubiq regime. Notably, under these simulation conditions the d-Phe residue of PMB1 was involved in just ∼6.7% of residue interactions; corresponding to the lowest percentage contribution of this residue to the binding of PMB1 to BLP across any simulation regime and ∼8.6% less than its contribution in the neutralized Ubiq regime. The Leu/d-Phe/DAB5 triad accounted for a combined ∼30.1% of observed residue interactions in this regime, corresponding to the lowest percentage involvement of this triad in any of the PMB1 simulation regimes. The polar residues of the branched fatty acid tail were involved in ∼30.6% of residue interactions, lower than was observed in the neutralized Ubiq regime (∼38.1%), yet considerably higher than the results from the concentrated Poly (∼17.6%) and Osmo (∼8.8%) regimes, providing further indication that under the crowded, chemically complex conditions of the Ubiq regime, this region plays an important role in the binding of PMB1 to BLP. Furthermore, the ∼28.0% contribution from the hydrophobic acyl tail of PMB1 corresponds to the highest percentage involvement of this residue in interactions with BLP across all simulation regimes. These results therefore indicate that, under these simulation conditions, the binding of PMB1 to BLP is underpinned primarily (∼88.7%) by interactions involving the amphipathic branched fatty acid tail and the Leu/d-Phe/DAB5 triad region of PMB1, albeit with a diminished contribution from the d-Phe residue itself.

### PME

Under neutralized conditions in the Poly and Osmo crowding regimes, no interactions were observed between PME and BLP. In contrast, in the higher salt conditions, such interactions were abundant, with 20 unique instances of PME-BLP binding observed in the Poly regime and 44 in the Osmo regime. Similar to PMB1, these interactions showed a strong preference for short durations, with median binding durations of 1.03 and 0.78 ns in the Poly and Osmo regimes, respectively. In the neutralized Ubiq regime, 64 unique instances of PME-BLP binding were observed, over double the number observed in equivalent simulations of PMB1. However, 63 of these binding events resulted from just two PME molecules that formed repeating transient interactions with BLP over the course of separate replicate simulations. Under these conditions, PME-BLP interactions were invariably brief with a median duration of 0.78 ns, and contrary to equivalent PMB1 simulations, there were no instances of PME molecules remaining bound to BLP for the duration of simulation. In the concentrated Ubiq regime, 66 unique instances of PME-BLP binding were observed, involving 8 individual PME molecules, exhibiting a median duration of 1.15 ns and no instances of PME molecules remaining bound to BLP for the entire simulation duration. Indeed, only one instance of a PME molecule remaining bound to BLP for the entire duration of the simulation was observed across all simulation regimes, occurring in the concentrated Osmo regime.

The binding of PME to BLP was largely dependent on polar interactions, accounting for ∼63.8% of residue interactions across all regimes. While these interactions predominantly involved the cationic DAB residues of PME, the Thr residues of PME accounted for ∼29.7% of the observed polar interactions, closely matching the value calculated from the simulations of PMB1. Similar to PMB1, the Ser residues of BLP were seen to interact with both the DAB and Thr residues of PME. Such interactions, however, accounted for only ∼9.1% of the total number of residue interactions, a lower percentage than was observed for the Gln (∼17.6%), Asp (∼15.6%), Ala (∼14.8%), Thr (∼14.7%), Asn (∼12.3%), and Lys (∼9.3%) residues of BLP. These results indicate that, similar to PMB1, the complete picture of PME-BLP binding is a balance of both electrostatic and hydrophobic interactions, involving many different residues.

In the concentrated Poly regime, polar interactions involving the DAB or Thr residues of PME were the dominant component in binding with BLP, making up ∼59.3% of all observed residue interactions. The Thr1 and DAB1 residues were of particular importance, contributing to, respectively, ∼17.9 and ∼15.7% of observed residue interactions. Notably, if we cluster the results according to the location of each residue in the structure of PME, we find that the polar DAB1/Thr1/DAB2 residues of the branched fatty acid tail accounted for a combined ∼41% of all observed residue interactions. The hydrophobic acyl tail of PME was involved in a further ∼25.3%, indicating that the complete branched fatty acid tail of PME plays a substantial role (∼66.3%) in the binding of PME to BLP under these simulation conditions.

This result is somewhat surprising since the heptapeptide ring of PME is much bulkier than its branched fatty acid tail and has a greater number of polar/charged residues. The apparent dominance of polar interactions on the binding of PME to BLP in this regime is therefore not distributed equally among all polar residues; rather, it is concentrated within the fatty acid tail region. Indeed, when compared to the results obtained from the equivalent PMB1 concentrated Poly regime, the greatest decrease in the percentage contribution of polymyxin residues is exhibited by the DAB3 (−10.0%) and Thr2 (−12.1%) residues on the heptapeptide ring of PME. Furthermore, the Leu1 and Leu2 residues of PME are responsible for, respectively, ∼6.2% and ∼4.1% fewer of the observed residue interactions than the equivalent Leu and d-Phe residues of PMB1. These data highlight that in the concentrated Poly regime the variety of polar and hydrophobic interactions that the heptapeptide ring of PMB1 experiences with BLP are diminished in the binding of PME to BLP.

In the concentrated Osmo regime, polar interactions involving the various DAB and Thr residues of PME dominated the binding of PME to BLP, accounting for a combined ∼79.9% of observed residue interactions. The DAB2, DAB5, and Thr1 residues of PME were of particular importance, accounting for, respectively, ∼26.3%, ∼ 22.9%, and ∼18.5% of observed residue interactions. The hydrophobic residues of PME were responsible for the remaining ∼20.1%, with the Leu1, Leu2, and acyl tail residues accounting for, respectively, ∼8.3%, ∼6.0%, and ∼5.9%.

Clustering the results based on the location of each residue within the structure of PME highlights that the polar residues of the branched fatty acid tail accounted for a combined ∼50.5% of observed residue interactions. The Leu1/Leu2/DAB5 triad in PME was responsible for a further ∼37.1% of observed residue interactions, just over half of the contribution from the analogous Leu/d-Phe/DAB5 triad in the equivalent PMB1 Osmo regime. While the importance of this triad was diminished in PME as compared to PMB1, the combination of its contribution with that of the DAB1/Thr1/DAB2 residues accounted for a combined ∼87.6% of observed residue interactions with BLP. These data therefore indicate that, in this regime, the interaction of PME with BLP is underpinned by interactions involving the polar region of the branched tail of PME and the Leu1/Leu2/DAB5 triad (Figure 6B).

The proportion of residue interactions that involved the hydrophobic residues of PME in the concentrated Osmo regime (∼20.1%) was approximately half that of the concentrated Poly regime (∼40.7%). This decrease may largely be attributed to the decreased involvement of the acyl tail of PME; this residue was responsible for ∼25.3% of residue interactions in the Poly regime compared to just ∼5.9% in the Osmo regime. Particular interest may be found in this result when compared to the previously described increase in the prevalence of hydrophobic interactions during the binding of PMB1 to BLP upon the inclusion of osmolytes, indicating that the presence of these osmolytes had an opposing effect on the biochemical nature of BLP binding with each type of polymyxin. However, given that the d-Phe residue of PMB1 accounted for 31.3% of all observed residue interactions in the concentrated Osmo regime, it is of no surprise that the substitution of this moiety for a Leucine residue in PME is accompanied by a markedly different distribution of residue interactions in this regime (Figure 7D,E).

In the neutralized Ubiq regime, just two PME molecules were responsible for all but one observed instance of PME-BLP binding. Both PME molecules were bound to clusters of ubiquitin proteins that were bound to the cell wall in close proximity to BLP. Neither PME molecule was observed to dissociate from these clusters, and further, the clusters did not dissociate from the cell wall. The two PME molecules thus remained in close proximity to BLP for the entire duration of simulation, forming repeated, transient interactions involving only those residues that were not already bound to the ubiquitin clusters. This behavior gives rise to the apparent dominance of the Thr1 (∼46.8%) and DAB3 (∼37.3%) residues of PME in binding with BLP under these simulation conditions.

In the concentrated Ubiq regime, polar interactions involving the various DAB and Thr residues of PME accounted for a combined ∼50.5% of observed residue interactions, ∼29.4% lower than the contribution of these same residues in the concentrated Osmo regime. Of these polar residues, the Thr1 and DAB1 residues of PME were of particular importance, contributing to, respectively, ∼16.0% and ∼12.7% of observed residue interactions. The hydrophobic residues of PME accounted for the remaining ∼49.5% of observed residue interactions, with the acyl tail, Leu2, and Leu1 residues accounting for, respectively, ∼30.4%, ∼11.8%, and ∼7.4%.

Clustering these results as before highlights that ∼65.7% of observed residue interactions involved residues located on the branched fatty acid tail of PME, with a further ∼19.2% arising from interactions involving the two hydrophobic Leu residues on the PME heptapeptide ring. The mean contribution of each remaining polar residue on the heptapeptide ring was just 3.8 ± 1.7%. The binding of PME to BLP under these simulation conditions is thus underpinned primarily (∼84.9%) by interactions involving the branched fatty acid tail and hydrophobic Leu residues of PME. This result is comparable to that obtained from the equivalent simulations of PMB1 in the concentrated Ubiq regime, in which PMB1-BLP binding was dominated by interactions involving the branched fatty acid tail and Leu/d-Phe/DAB5 triad, the latter of which, excluding the DAB5 residue, is positionally analogous to the two Leu residues of PME.

## Discussion

To date, mechanistic studies of polymyxin action have focused almost entirely on the two membranes of Gram-negative bacteria;^59,60^ leaving unaddressed the question of how polymyxins cross the periplasm from their point of entry into the cell, the OM, to reach the target of their antimicrobial action, the IM. Here, we have simulated an all-atom model of the *E. coli* cell envelope under various levels of biomolecular crowding to investigate the molecular interactions of PMB1 and PME as a function of environmental complexity. We have shown that both PMB1 and PME interact with the cell wall predominantly via polar interactions between the cationic DAB residues of the polymyxins and the carboxylate groups of the meso-DAP, d-Glu, and d-Ala residues of the cell wall peptide stems in all environments (albeit to different extents). In the absence of other biochemical species (osmolytes or proteins), this interaction is rapidly formed and leads to irreversible binding.

The dependency of polymyxin binding to the cell wall on this singular type of interaction presented an opportunity for cations and cationic moieties of other molecules within the more chemically diverse environments we simulated to disrupt the interaction between polymyxins and the cell wall. Indeed, it was found that the addition of physiological salt concentrations, osmolytes, and increased biomolecular crowding all acted to decrease the duration of binding between the polymyxins and the cell wall. However, dissociation of polymyxins from the cell wall was only observed in simulations with physiological salt concentrations or with lower salt concentrations but the presence of crowding ubiquitin proteins. These observations may all be well explained through the lens of this cationic disruption.

Since Cl^–^ ions were sufficient to neutralize our cell envelope model (in addition to the Ca^2+^ that neutralized and remained bound to LPS), it was only under excess salt concentrations that K^+^ ions were present in our simulations. These freely diffusing cations were observed to coordinate to cell wall carboxylate groups; this regularly occurred in close proximity to polymyxin molecules, resulting in direct competition for cell wall interaction sites. In this way, K^+^ ions disrupted the electrostatic interactions via which polymyxins otherwise bound to the cell wall, allowing for the dissociation of polymyxin molecules from the cell wall. In the most crowded simulation regime, in which polymyxin–cell wall dissociation was observed even in the absence of K^+^ ions (only neutralizing Cl^–^ ions were present), the addition of K^+^ further decreased the duration of polymyxin–cell wall binding. Thus, we find that K^+^ disrupts polymyxin–cell wall interactions under all environmental conditions.

These observations follow the established notion of the “salting-in” of proteins, whereby low (<0.2–0.5 M) salt concentrations lead to an increase in protein solubility.^61−63^ This increase in solubility is attributed to ions “coating” proteins in solution, screening the electrostatic interactions between neighboring proteins and increasing the relative activity of nearby solvent molecules. In our simulations, K^+^ ions were observed to preferentially aggregate at the cell wall surface throughout all crowding regimes (Figure S7), thus “coating” the cell wall. The resulting abundance of K^+^ coordination with the peptide residues of the cell wall screened the electrostatic interactions of these cell wall residues with nearby polymyxin molecules. We expect that this screening acted to decrease the strength of the electrostatic interaction between these polymyxin molecules and the cell wall, leading to a relative increase in the activity of nearby solvent molecules on the polymyxins, thus resulting in the observed increase in their solubility. The distribution of K^+^ throughout the periplasm remained unchanged during an extended simulation of the PMB1 concentrated Ubiq regime that was extended for an additional 250 ns starting from the output of one of the original replica trajectories.

Similarly, upon the addition of osmolytes to our simulations, spermidine was seen to interact with the same carboxylate groups of the meso-DAP, d-Glu, and d-Ala residues of the cell wall. Spermidine is a cationic polyamine and so it is of no surprise that it too would compete with the cationic DAB residues of the polymyxins for interaction sites on the cell wall. Indeed, similar to K^+^, we observed spermidine in direct competition with neighboring polymyxin molecules for cell wall interaction sites; however, the low concentration of spermidine within our simulations inherently limited our sampling of such events. Despite this, the number of contacts between cell wall oxygen atoms and spermidine was found to express a negative correlation with the number of contacts between cell wall oxygen atoms and the DAB residues of the polymyxins; indicating that despite our limited sampling, the presence of spermidine still imposed a measurable disruption to the binding of polymyxin molecules to the cell wall.

Finally, the ubiquitin proteins present in our most crowded envelope models were also seen to interact with the cell wall via polar interactions with the carboxylate groups on the meso-DAP, d-Glu, and d-Ala residues of the cell wall peptide stems. Ubiquitin is a large, nitrogen rich molecule with multiple cationic lysine and amine-rich arginine residues on its surface and it is these residues in particular that were seen to coordinate with the carboxylate groups on the cell wall. It was only in the presence of these ubiquitin proteins that polymyxins were seen to dissociate from the cell wall under neutralizing salt concentrations; and this result too can be understood in the context of cationic disruption.

Within the neutralized Poly regime, the polymyxins were the only freely diffusing cationic compounds within the periplasm and thus had no direct competition for cell wall interaction sites; concurrent with no observations of polymyxins dissociating from the cell wall under these conditions. In simulations of the neutralized Osmo regime, only a single cationic spermidine molecule was included in the system; thus, competition between spermidine and polymyxins for cell wall interaction sites was inherently limited and was again concurrent with no observations of polymyxins dissociating from the cell wall. In contrast to this, the neutralized Ubiq regime included 11 nitrogen-rich ubiquitin proteins, each with numerous surface lysine and arginine residues. The tendency for these residues to interact with the carboxylate groups on the cell wall peptide stems, along with the large excluded volume effects resulting from multiple such proteins binding to the cell wall simultaneously, represented the greatest competition for cell wall interaction sites faced by the polymyxins in any of the neutralized simulation regimes. These behaviors limited the ability of polymyxins to form long-lasting interactions with the cell wall and led to the abundant dissociation of polymyxin molecules from the cell wall.

Our analysis of polymyxin interactions with BLP highlighted a different picture of how environmental complexity impacts protein-peptide interactions within the cell envelope. While the interactions between the polymyxins and BLP exhibited a preference for short durations across all simulation regimes, the residue interactions that were observed to underpin their binding were dependent both on polymyxin type and system complexity. This is in stark contrast to the effects that increasing complexity had on polymyxin–cell wall binding, wherein we observed the binding durations to decrease while the underlying residue interactions remained consistent.

It was previously reported that the serine and acidic residues of BLP had a particular propensity to interact with PMB1;^26^ however, we have shown here that it is in fact a multitude of both polar and hydrophobic interactions that give rise to the binding of both PMB1 and PME to BLP. The balance of these polar and hydrophobic interactions was similar for both polymyxins in the Poly regime; however, deviations emerged in the Osmo and Ubiq regimes, exemplified by the concentrated Osmo regime, in which PMB1-BLP binding was dominated by hydrophobic interactions and PME-BLP binding was dominated by electrostatic interactions involving cationic DAB residues. Our analysis indicated that, under these conditions, almost a third of all PMB1-BLP residue interactions involved the hydrophobic d-Phe residue of PMB1; since this moiety is substituted for a d-Leu residue in PME, the deviation in residue interaction distributions between the two polymyxin species is unsurprising, albeit unexplained as of yet.

Despite the deviations observed between simulation regimes and polymyxin species, we have highlighted how certain groups of residues in both PMB1 and PME are repeatedly found to play critical roles in binding with BLP under different simulation conditions. In particular, the positionally analogous Leu/d-Phe/DAB5 and Leu1/Leu2/DAB5 triads of PMB1 and PME, respectively, as well as the polar DAB1/Thr1/DAB2 triad of the polymyxin branched fatty acid tail were of repeated importance.

The exact mechanisms by which the structural differences between PMB1 and PME, along with any underlying molecular interactions with the various ions, osmolytes, and proteins in our system, may give rise to the regime-dependent variations in the residue interaction distributions for each individual polymyxin species are not well understood as of yet, leaving open a target for future work to further our understanding of the challenges posed to the motion of the polymyxins as they traverse the bacterial cell envelope.

A recent experimental study^64^ highlighted that the diffusion of a protein, OsmY, throughout the *E. coli* periplasm was best described by a two-component random walk model, comprising one fast and one slow diffusive component. The presence of the putative slow diffusion component implied that a fraction of the OsmY proteins interacted with the various supramolecular structures within the periplasm, limiting their diffusion rate. In contrast, the fast diffusion component describes those proteins that were freely diffusing throughout the periplasm. The diffusion coefficient of the fast diffusion component was found to increase with periplasmic volume, implying that the free diffusion rate of proteins within the periplasm was negatively correlated with the extent of macromolecular crowding within the surrounding environment.

Our observations of sustained complex formation between the polymyxins and both BLP and the cell wall support the notion that interactions with the various surfaces of the *E. coli* periplasm limit the diffusion rates of proteins within this environment. We propose, however, that the extent to which such interactions, particularly with the cell wall, disrupt the diffusion of the polymyxins is modulated by the presence of cations, such as K^+^, within the environment. We also note here that this modulation, whereby K^+^ enable the free diffusion of polymyxins throughout the periplasm, may enable the polymyxins to more readily encounter lipoprotein carriers such as LolA, which have been previously proposed to provide polymyxins with a potential passive transport mechanism within the periplasm.^26^

Furthermore, we have provided qualitative evidence that the inclusion of crowding ubiquitin proteins restricted the lateral and vertical diffusion of polymyxin molecules throughout the bulk aqueous phase of the periplasm, thus providing support for the negative correlation between the free diffusion rate of proteins and macromolecular crowding within the periplasm.

## Conclusion

Overall, it is clear that the pattern of molecular interactions within the periplasm is highly convoluted. We have only scratched the surface, as there are many more molecular species present in the periplasm than it is feasible for us to simulate here on statistically significant time scales. However, our simulations have certainly highlighted that the patterns and modes of interaction of lipoprotein antibiotics with the cell wall and proteins within the periplasm are impacted by the nature of the other species present in the vicinity. The search for polymyxin derivatives is an active area,^65^ and it is our contention that a more nuanced consideration of the local environment may be beneficial for this end and, indeed, more generally for the future design of antibiotics that negotiate the periplasm to either act on the inner membrane or penetrate beyond the inner membrane into the cytoplasm.

## Supporting Information Available

Tables containing residue interaction percentage data for interactions between PMB1-PGN, PME-PGN, PMB1-BLP, and PME-BLP; hydrogen bond count between water molecules and the cell wall in the Poly and Ubiq regimes; minimum distances between interacting residues of the cell wall and PMB1/potassium during direct competition; minimum distances between interacting residues of the cell wall and PMB1/spermidine during direct competition; visualization of spermidine insertion into clustered junction region of the cell wall; PMB1 lateral and vertical diffusion under replacement of K^+^ for Ca^2+^; interaction of PMB1 with BLP via the Leu/d-Phe/DAB5 residue triad; vertical distribution of K^+^ (or Ca^2+^) ions in each simulation regime (PDF)
