# Supplementary material for: Molecular Crowding Alters the Interactions of Polymyxin Lipopeptides within the Periplasm of E. coli: Insights from Molecular Dynamics
Source: J Phys Chem B. 2024 Mar 8;128(11):2717–33. doi: 10.1021/acs.jpcb.3c07985 (PMC10961723; doi:10.1021/acs.jpcb.3c07985)
Supplement: Supplementary file 1 — jp3c07985_si_001.pdf [file jp3c07985_si_001.pdf]

Molecular crowding alters the interactions of polymyxin lipopeptides within the periplasm of *E. coli:* insights from molecular dynamics.

Iain P. S. Smith, Conrado Pedebos, Syma Khalid

Supporting Information

Table S1: PMB1-PGN residue interaction percentages

**Residues**		**Simulation Regime**											
**PGN**	**PMB**	**poly-neut**		**poly-conc**		**osmo-neut**		**osmo-conc**		**ubiq-neut**		**ubiq-conc**	
m-DAP	DAB1	31.21	18.44	34.49	16.67	31.75	14.02	33.83	14.78	35.51	18.04	30.48	13.63
D-Glu	DAB2	21.65	19.51	22.51	17.18	21.95	20.93	22.7	19.34	21.97	19.28	22.79	21.59
D-Ala	DAB3	18.3	15.01	20.23	15.89	18.64	15.68	18.43	16.81	19.28	15.38	17.45	19.55
L-Ala	DAB4	7.51	15.24	8.57	18.78	9.08	18.76	9.88	17.22	6.56	20.39	6.75	22.46
GlcNAc	DAB5	8.36	17.68	5.84	15.86	7.35	15.06	6.53	16.28	6.66	12.87	7.52	9.88
MurNAc	Thr1	12.97	3.2	8.35	3.55	11.22	3.23	8.62	2.85	10.02	4.45	15.01	2.57
	Thr2		3.41		3.49		3.25		2.83		3.02		3.58
	Acyl Tail		3.63		3.49		2.55		3.59		1.25		2.87
	D-Phe		2.09		2.78		4.12		3.36		2.6		1.99
	Leu		1.79		2.31		2.41		2.94		2.72		1.89
	hydrophobic	7.51		8.57		9.08		9.88		6.56		6.75	
	DAB	85.88		84.38		84.44		84.43		85.97		87.1	
	Thr	6.61		7.04		6.48		5.68		7.47		6.15	

Table S2: PME-PGN residue interaction percentages

**Residues**		**Simulation Regime**											
**PGN**	**PME**	**poly-neut**		**poly-conc**		**osmo-neut**		**osmo-conc**		**ubiq-neut**		**ubiq-conc**	
m-DAP	DAB1	28.76	15.02	34.77	17.93	30.08	14.85	30.81	15.04	30.81	15.74	36.04	17.45
D-Glu	DAB2	22.07	22.01	22.19	16.13	24.2	17.84	24.21	16.78	22.75	18.66	22.31	17.84
D-Ala	DAB3	19.36	20.56	19.55	18.47	16.18	22.05	19.66	20.23	18.05	19.77	20.25	20.01
L-Ala	DAB4	5.71	15.35	8.54	15.64	7.91	17.88	9.69	17.47	7.47	18.53	6.56	16.27
GlcNAc	DAB5	10.14	16.51	5.71	16.31	9.71	13.67	5.96	15.07	8.85	14.84	4.16	16.74
MurNAc	Thr1	13.96	2.93	9.24	3	11.92	2.74	9.67	2.57	12.06	2.61	10.68	2.06
	Thr2		1.91		3.98		3.06		3.15		2.37		3.07
	Acyl Tail		3.1		3.64		2.63		3.78		2.14		2.53
	Leu1		1.04		1.89		2.34		2.89		3.02		1.63
	Leu2		1.57		3.01		2.95		3.02		2.31		2.39
	hydrophobic	5.71		8.54		7.91		9.69		7.47		6.56	
	DAB	89.45		84.47		86.29		84.59		87.54		88.31	
	Thr	4.84		6.98		5.8		5.72		4.98		5.13	

Table S3: PMB1-BLP residue interaction percentages

Residues		Simulation Regime									
**BLP**	**PMB**	**poly-neut**		**poly-conc**		**osmo-conc**		**ubiq-neut**		**ubiq-conc**	
ASP	DAB1	21.18	2.82	14.84	8.1	13.32	2.25	11.05	9.07	27.2	17.52
THR	DAB2	2.82	12.94	12.1	5.77	6.66	3.32	1.98	8.95	6.66	3.03
GLN	DAB3	18.35	11.29	21.34	13.22	6.89	1.56	22.78	4.35	8.74	1.71
SER	DAB4	1.41	5.88	10.29	5.66	3.69	1.52	8.14	0.13	3.67	9.32
ALA	DAB5	28.24	1.41	18.84	9.55	14.72	22.6	8.62	19.4	16.09	10.12
LYS	Thr1	0	4	7.31	3.68	9.91	3.22	9.55	20.12	8.29	10.02
ASN	Thr2	2.12	8.94	5.39	13.1	8.43	0.81	18.08	0.38	4.7	0.28
ARG	Acyl Tail	25.88	0	6.65	15.15	20.08	22.97	8.8	13.12	22.7	28.03
LEU	D-Phe	0	26.82	1.51	15.76	0.42	31.31	5.77	15.26	0.28	6.67
VAL	Leu	0	25.88	0.59	10.02	8.59	10.45	0.49	9.23	1.66	13.29
ILE		0		0.71		0		4.69		0	
MET		0		0.42		7.3		0.04		0	
TYR		0		0		0		0		0	
	hydrophobic	52.71		40.92		64.73		37.61		48	
	DAB	34.35		42.3		31.24		41.9		41.7	
	Thr	12.94		16.78		4.03		20.5		10.3	

Table S4: PME-BLP residue interaction percentages

Residues		Simulation Regime							
**BLP**	**PME**	**poly-conc**		**osmo-conc**		**ubiq-neut**		**ubiq-conc**	
ASP	DAB1	21.07	15.74	17.57	5.68	25.92	9.25	11.01	12.65
THR	DAB2	3.34	7.35	6.59	26.28	24	0.79	27.27	6.71
GLN	DAB3	18.63	3.22	20.87	2.93	9.51	46.77	14.24	5.12
SER	DAB4	4.75	4.63	13.5	2.07	0	0	7.41	4.9
ALA	DAB5	21.03	9.41	9.19	22.87	0	0	17.37	3.59
LYS	Thr1	17.36	17.9	3.42	18.52	0	37.26	11.09	15.99
ASN	Thr2	5.8	0.99	25.2	1.59	11.61	3.75	3.51	1.5
ARG	Acyl Tail	8	25.28	2.83	5.86	28.97	2.18	7.93	30.38
LEU	Leu1	0.01	3.81	0.49	8.26	0	0	0.12	7.38
VAL	Leu2	0.01	11.66	0.07	5.96	0	0	0.02	11.78
ILE		0		0.26		0		0	
MET		0		0		0		0	
TYR		0		0.01		0		0.03	
	hydrophobic	40.75		20.07		2.18		49.54	
	DAB	40.36		59.82		56.81		32.97	
	Thr	18.89		20.11		41.01		17.49	

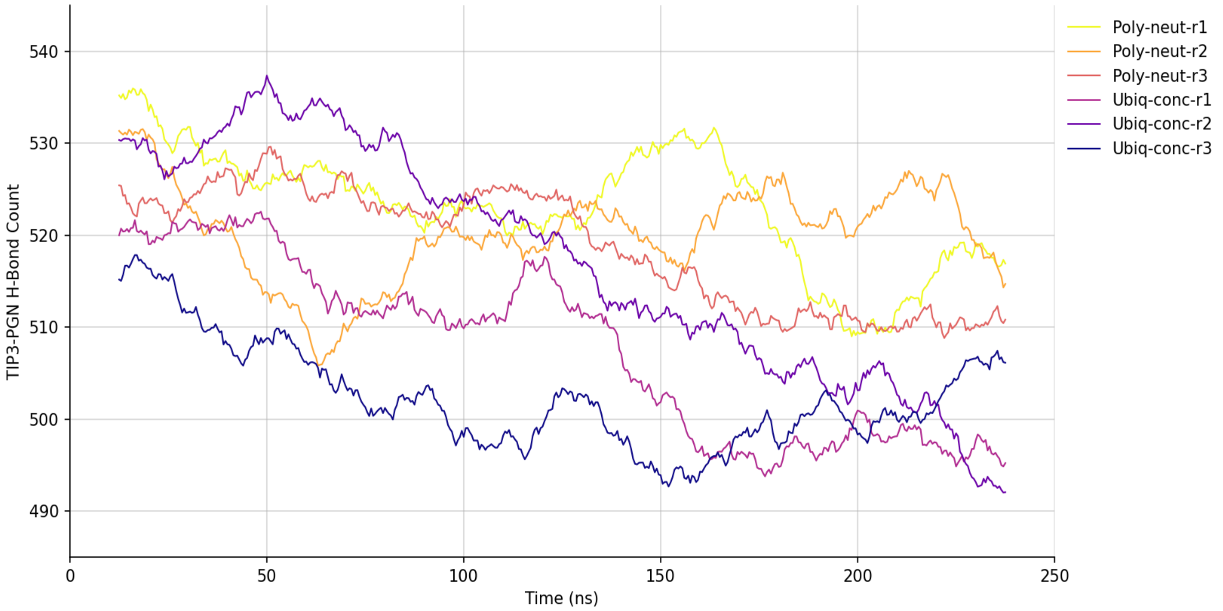

Figure S1) Number of hydrogen bonds formed between water molecules (TIP3) and the cell wall (PGN) throughout each replicate simulation of the neutralised Poly and concentrated Osmo regimes in the presence of PMB1. Data is plotted as a moving average across 25 ns windows.

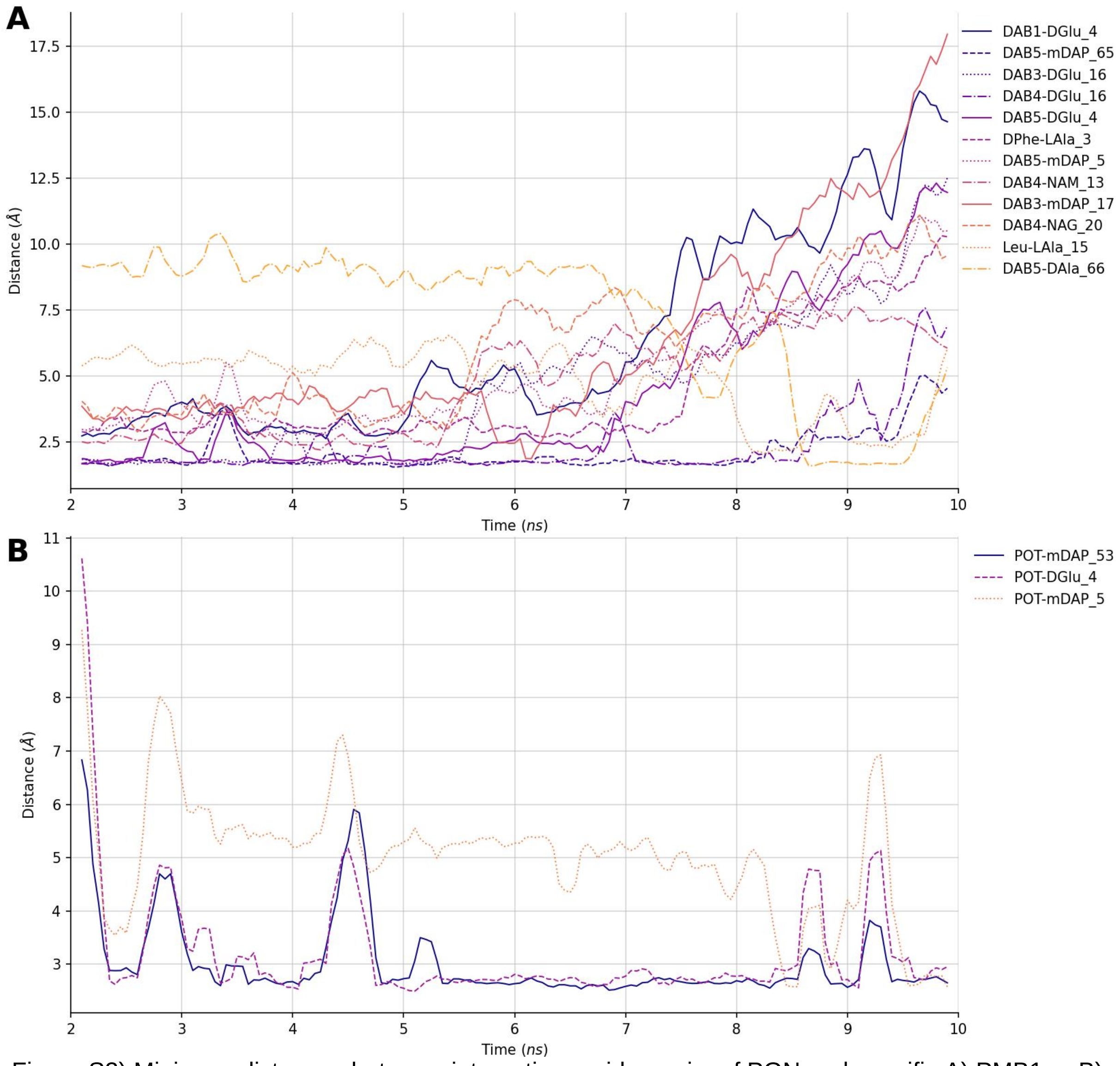

Figure S2) Minimum distances between interacting residue pairs of PGN and specific A) PMB1 or B) potassium moieties observed in competition with each other for cell wall interaction sites (Figure 3A). Data plotted for the time during which PMB1/potassium were within ~3 nm of each other. Residue names on the left correspond to A) PMB1 / B) potassium, residue names on the right correspond to PGN.

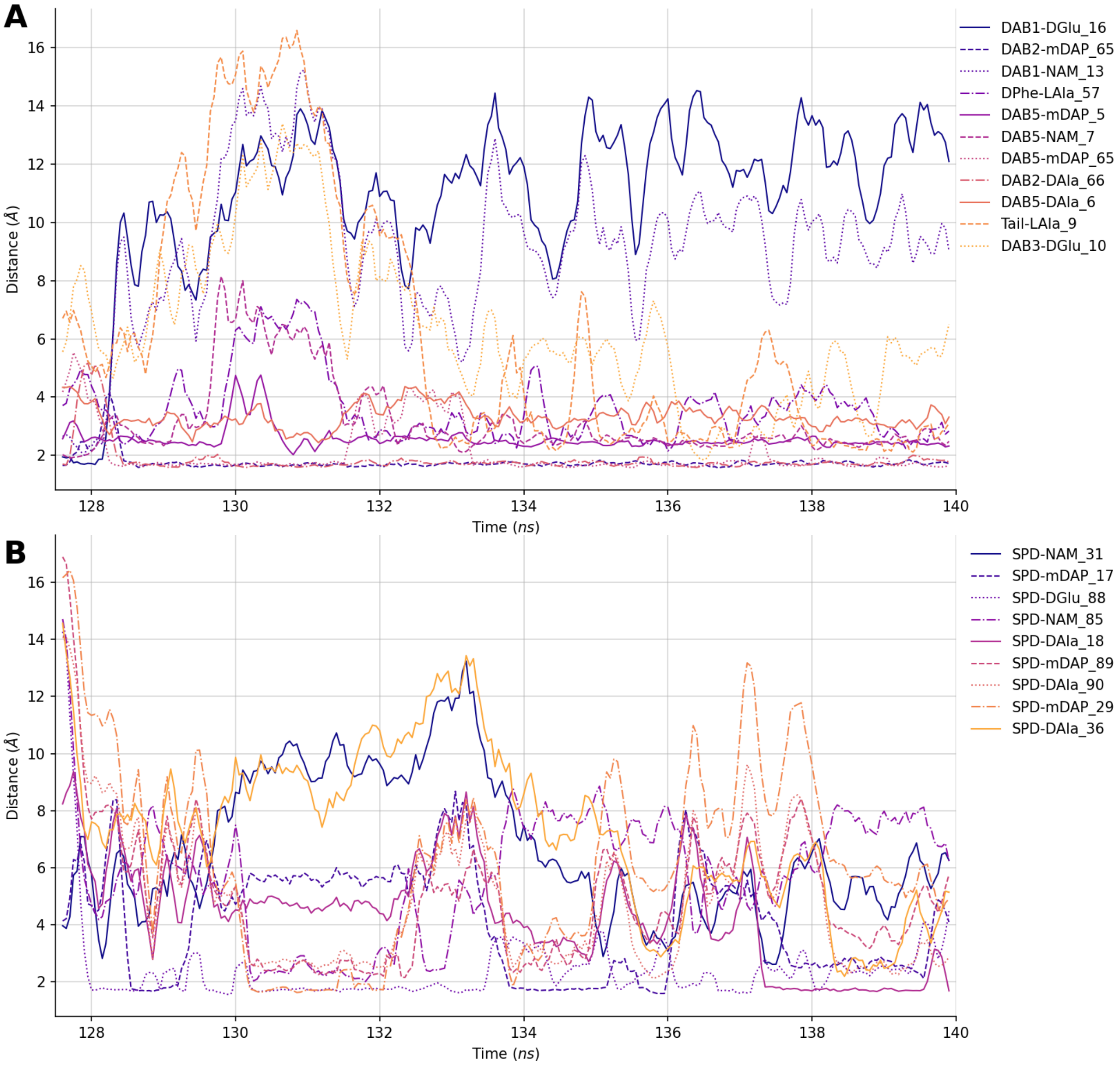

Figure S3) Minimum distances between interacting residue pairs of PGN and specific A) PMB1 or B) spermidine moieties observed in competition with each other for cell wall interaction sites (Figure 4A). Data plotted for the time during which PMB1/spermidine were within ~3 nm of each other. Residue names on the left correspond to A) PMB1 / B) SPD, residue names on the right correspond to PGN.

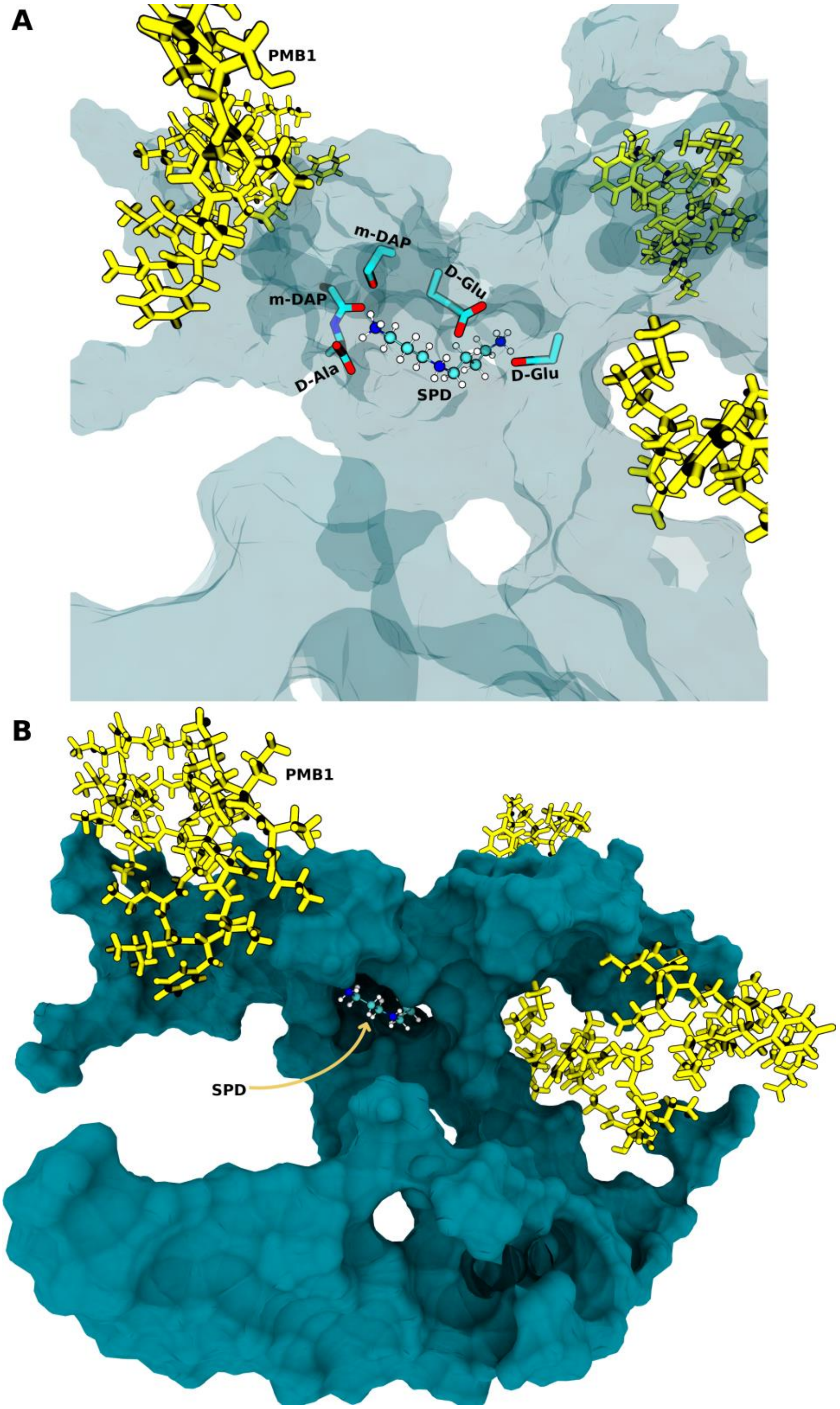

Figure S4) Insertion of spermidine into clustered junction region of PGN cell wall, snapshots taken from the neutralised Osmo regime. A) Orthographic view with specific residues of PGN interacting with SPD highlighted in licorice. B) Perspective view highlighting the cell wall cavity into which SPD is inserted. The cell wall is represented by the transparent (A) or diffuse (B) cyan surface plot.

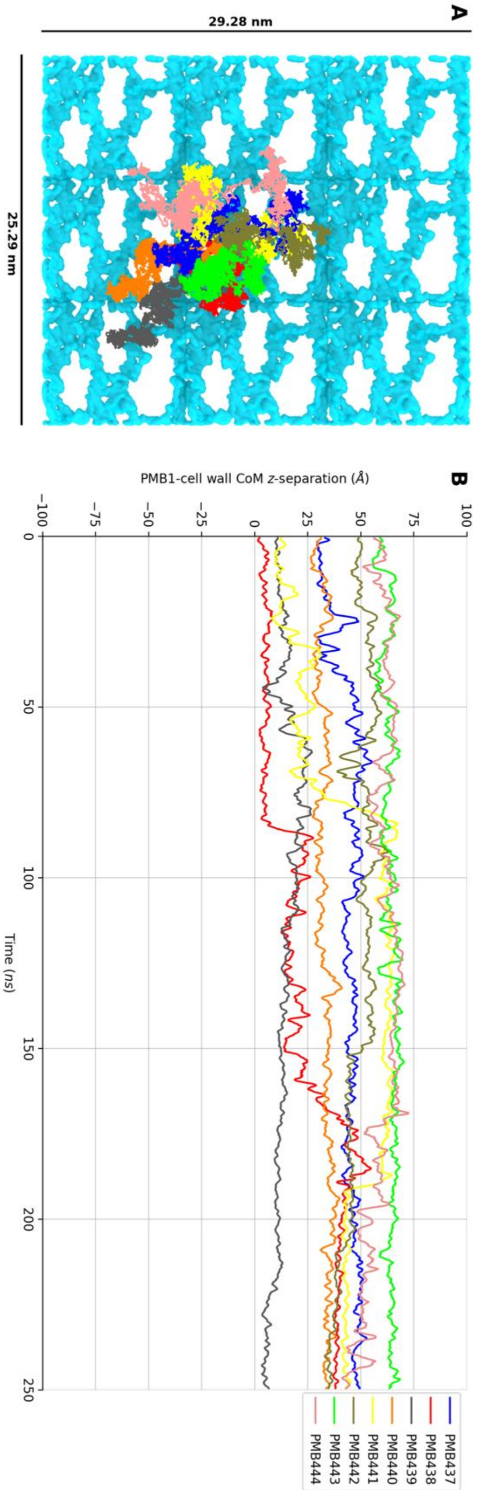

Figure S5) PMB1 diffusion in the simulation of the concentrated Ubiq regime under the replacement of K^+ ions for Ca^{2+}. A) Trajectories of each PMB1 molecule in the x-y plane. B) CoM z-coordinates of each PMB1 throughout the simulation.

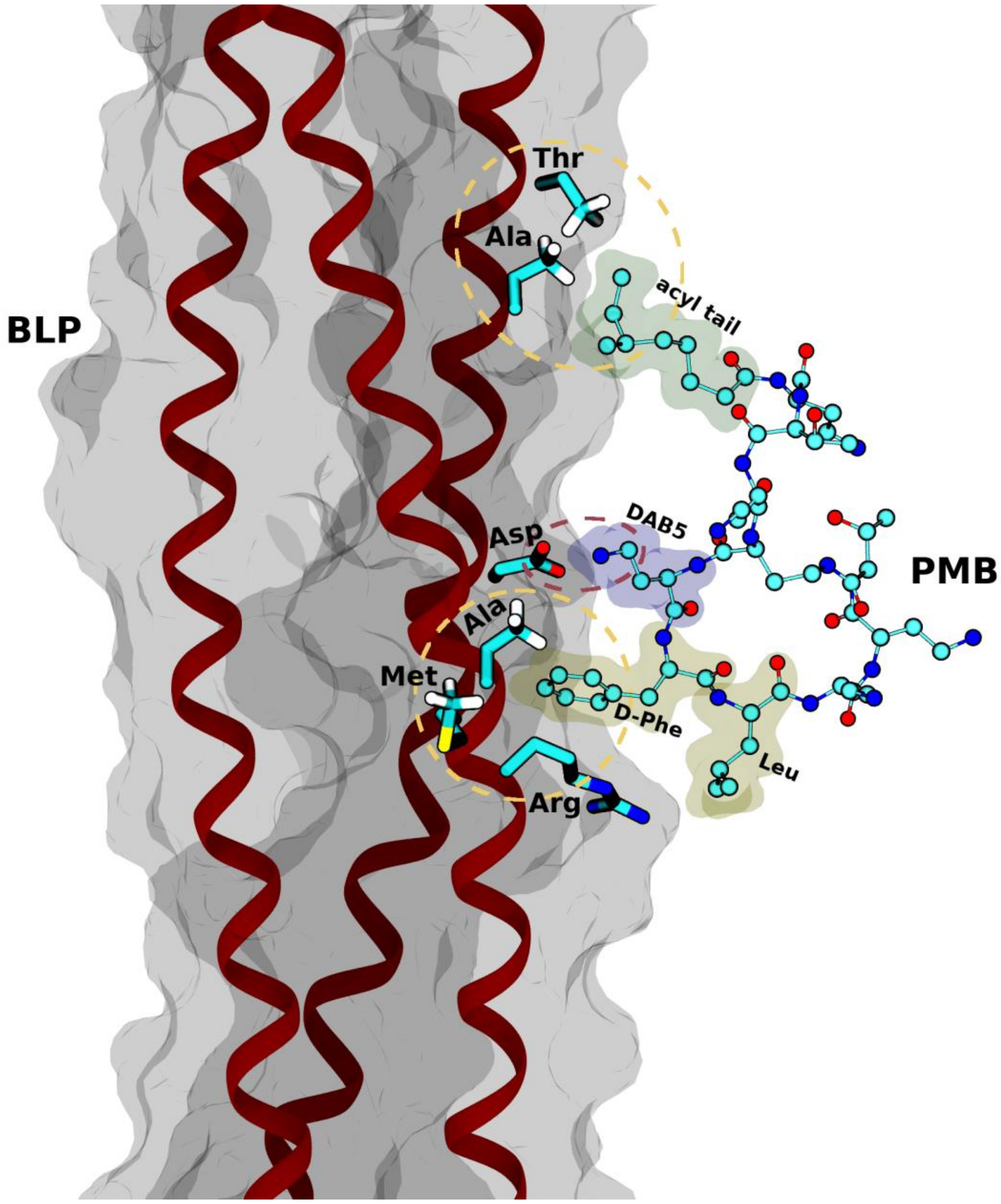

Figure S6) PMB1 bound to BLP in the concentrated Osmo regime via interactions involving the Leu/D-Phe/DAB5 triad and acyl tail of PMB1 with various residues of BLP. Yellow dashed circles highlight clustering of hydrophobic residues, red dashed circle highlights salt bridge formation between charged DAB5/Asp residues of PMB1/BLP.

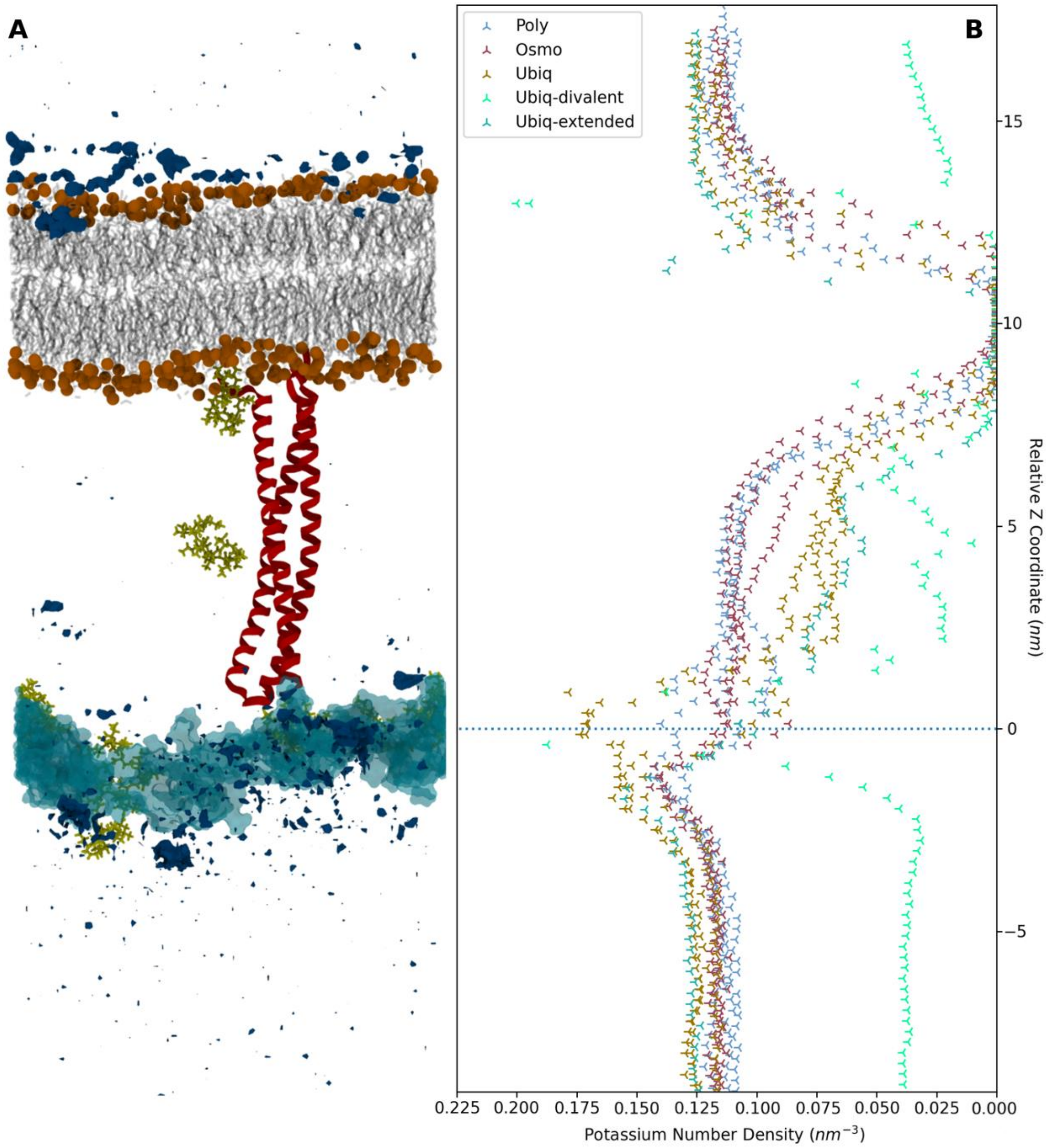

Figure S7. A) Representative average volume map of potassium ions (dark blue) calculated across one replica of the concentrated Poly regime. PMB1 represented in yellow, BLP in red ribbons, PGN in transparent cyan, OM headgroup phosphates in orange vdW. B) Partial z-densities of K^+ (Ca^{2+} in divalent simulation) across all simulations. Z-coordinates are measured relative to the cell wall CoM.
